# Psychobehavioral profiles, competitive progression, and nutritional characteristics in CrossFit® athletes across a national competitive season

**DOI:** 10.3389/fspor.2026.1902121

**Published:** 2026-10-06

**Authors:** Gabriel de Souza Zanini, Tatiana Palotta Minari, Eduarda Cortese Santos, Víctor Hernández-Beltrán, José M. Gamonales, David Michel de Oliveira, Cassiano Merussi Neiva, Mário Cunha Espada, Dalton Müller Pessôa Filho

**Affiliations:** 1 Post-Graduate Program in Movement Science, São Paulo State University (UNESP), Bauru, Brazil; 2 Department of Physical Education, Faculdades Integradas de Jaú (FIJ), Fundação Educacional “Dr. Raul Bauab” - Jahu, Jaú, Brazil; 3 Department of Bioscience, Federal University of São Paulo (UNIFESP), Santos, Brazil; 4 Training Optimization and Sports Performance Research Group (GOERD), Faculty of Sport Science, University of Extremadura, Cáceres, Spain; 5 Faculty of Education and Psychology, University of Extremadura, Badajoz, Spain; 6 Instituto Universitario de Investigación e Innovación en el Deporte (INIDE), University of Extremadura, Cáceres, Spain; 7 Graduate Program in Biosciences and One Health, Institute of Biosciences (IB); Graduate Program in Health Sciences, Institute of Health Sciences (IHS), Federal University of Jataí (UFJ), Jataí, Brazil; 8 Post-graduate Program in Human Development and Technology, São Paulo State University (UNESP), Rio Claro, Brazil; 9 Escola Superior de Educação, Instituto Politécnico de Setúbal, Setúbal, Portugal; 10 Sport Physical Activity and Health Research & Innovation Center (SPRINT), Sport Sciences School of Rio Maior (ESDRM), Instituto Politécnico Santarém, Rio Maior, Portugal; 11 Faculdade de Motricidade Humana, Centro Interdisciplinar de Performance Humana (CIPER), Universidade de Lisboa, Lisbon, Portugal; 12 Comprehensive Health Research Centre (CHRC), Universidade de Évora, Évora, Portugal

**Keywords:** athletic performance, competitive anxiety, crossFit ®, high-intensity functional training, mood states, prospective observational study, psychobehavioral monitoring, stress and recovery

## Abstract

**Background:**

Competitive CrossFit® involves substantial physiological, psychological, recovery-related, and nutritional demands throughout a competitive season. However, prospective field-based investigations examining psychobehavioral characteristics across successive competitive stages while simultaneously characterizing the broader training and nutritional context of competitive athletes remain limited. This study primarily investigated psychobehavioral and recovery-related profiles across competitive stages and their associations with subsequent competitive progression during a national CrossFit® season in Brazil. As a secondary exploratory objective, training, anthropometric, and self-reported nutritional characteristics were examined within the same real-world competitive setting.

**Methods:**

A prospective observational study followed an initial cohort of 239 CrossFit® athletes across three successive competitive stages of the 2025 TCB season. Athletes were followed across the championship's progressive competitive structure, comprising the 4ALL online qualifier, the in-person Super Selective events, and the national Finals. Mood states were assessed using the Brunel Mood Scale (BRUMS), perceived stress and recovery using the RESTQ-Sport, and state anxiety using the STAI-State. Psychobehavioral analyses examined differences across competitive stages and associations with subsequent progression, whereas training, anthropometric, and nutritional variables were treated as complementary contextual and exploratory characteristics.

**Results:**

Athletes progressing to later competitive stages demonstrated lower scores for tension, depression, fatigue, confusion, total mood disturbance (TMD), stress, and anxiety, together with higher vigor and recovery scores. The largest stage-related effect was identified for TMD (*χ*^2^ = 184.311; marginal *R*^2^ = 0.501). Competitive category demonstrated stronger interaction effects than sex for psychobehavioral variables. Baseline analyses showed that semifinalists and finalists exhibited more favorable psychological profiles than eliminated athletes. Multinomial logistic regression demonstrated that lower anxiety, lower TMD, lower global stress, and higher global recovery were associated with greater odds of competitive progression. Nutritional analyses showed low explanatory capacity for BMI, with no significant associations between carbohydrate, protein, or fat intake and BMI in the exploratory regression model.

**Conclusion:**

Athletes who advanced to later competitive stages showed more favorable psychobehavioral and recovery-related profiles, characterized by lower anxiety, mood disturbance, and stress, and higher perceived recovery. These variables were associated with competitive progression in this cohort, suggesting their potential usefulness for athlete monitoring during CrossFit® competitive seasons. However, given the observational design and the elimination-based structure of the competition, these findings should be interpreted as associations rather than causal effects.

## 1 Introduction

Over the past decades, CrossFit® has emerged as a prominent high-intensity functional training modality characterized by highly varied functional tasks that, in competitive settings, can be broadly organized into weightlifting and throwing, gymnastics, and metabolic conditioning (1–5). Alongside its global expansion, CrossFit® has also established a well-structured competitive landscape, with progressive events such as the CrossFit® Games, the sport's annual world championship, as well as national and international competitions. These competitive environments expose athletes to high levels of physical and psychological demands throughout the season, placing them under conditions comparable to those experienced by athletes in traditional sports (4, 6–9).

In this context, competitive CrossFit® is characterized by complex and multifactorial demands, in which physiological, emotional, and nutritional factors continuously interact to determine sports performance (4, 10–12). From a physiological perspective, CrossFit® imposes substantial metabolic and neuromuscular stress, involving repeated high-intensity efforts, high variability of stimuli, and limited recovery intervals. These characteristics result in significant cardiovascular strain, metabolite accumulation, and elevated ratings of perceived exertion (4, 8, 13, 14).

Simultaneously, the emotional demands associated with the competitive environment play a critical role in performance. Variables such as mood states, anxiety, stress, and perceived recovery have been widely recognized as key modulators of performance across different sports contexts (6, 15–18), influencing both execution capacity and tolerance to training loads (19, 20). Evidence suggests that imbalances in these parameters, such as increased fatigue and tension combined with reduced vigor, are directly associated with performance decrements, increased injury risk, and the development of overreaching and overtraining states (21–23).

Nutrition also represents a relevant contextual factor in competitive CrossFit®. The high energetic and recovery demands of the modality require adequate energy and macronutrient availability to support substrate replenishment, muscle recovery, and repeated high-intensity training and competition demands (12, 24–27). Accordingly, nutritional practices may contribute to the broader physiological context in which athletes prepare for and perform during competition. However, competitive progression is inherently multifactorial, and nutritional status should not be considered an independent determinant of qualification. In the present study, dietary intake was therefore examined as a secondary exploratory characteristic of the competitive cohort rather than as a longitudinal predictor of competitive progression.

Despite the growing body of evidence on CrossFit®, the literature remains predominantly focused on acute physiological responses and physical performance indicators, with limited investigation into the longitudinal interaction between behavioral, emotional, and contextual variables throughout extended competitive periods. This gap is particularly relevant, as sports performance is the result of dynamic adaptive processes that depend on the interaction between training load, nutritional adequacy, recovery, and the athlete's psychobehavioral and recovery-related state over time. Therefore, longitudinal approaches integrating multiple dimensions are essential to achieve a more comprehensive understanding of performance determinants in competitive CrossFit®. Moreover, analyses conducted in real-world ecological contexts, such as full competitive seasons, enhance the applicability of findings for coaches, athletes, and practitioners.

Although recent reviews have mapped the physiological demands and nutritional evidence related to CrossFit®, much of the available literature remains centered on acute workouts, isolated performance outcomes, or cross-sectional descriptions (4, 24, 28). Prospective evidence obtained across complete competitive periods remains comparatively limited, particularly regarding how psychobehavioral status, perceived recovery, training background, and dietary characteristics are represented among athletes exposed to progressively more selective stages of competition. Real-world competitive investigations may therefore complement laboratory-based studies by preserving the interaction among athlete characteristics, competitive demands, and the changing context of the season, thereby providing a more comprehensive understanding of the factors associated with competitive progression in CrossFit®.

Within this perspective, two related but conceptually distinct questions are relevant. First, athletes represented at successive competitive stages may display different psychobehavioral and recovery-related profiles. Because advancement occurs through progressive elimination, such stage-related differences may reflect both characteristics already present among athletes who subsequently qualify and changes occurring among repeatedly assessed athletes. Second, baseline and stage-specific characteristics may be associated with the probability of subsequent competitive progression. Distinguishing these questions is essential because competitive stage should not be interpreted simultaneously as a purely temporal measure and as an unqualified proxy for competitive performance.

Therefore, the primary aim of the present study was to investigate psychobehavioral and recovery-related profiles across the successive stages of the 2025 Torneio CrossFit Brasil (TCB) and to examine whether these characteristics were associated with subsequent competitive progression. Specifically, mood states, state anxiety, perceived stress, and perceived recovery were evaluated across the 4ALL qualifier, Super Selective, and national Finals. As a secondary exploratory objective, the study characterized training, anthropometric, and self-reported nutritional characteristics of athletes within the same real-world competitive environment.

Based on previous evidence linking mood disturbance, anxiety, fatigue, and insufficient recovery with competitive demands and sport performance, we hypothesized that athletes who subsequently progressed to later competitive stages would present lower anxiety, lower mood disturbance and perceived stress, and higher perceived recovery. However, given the multifactorial nature of competitive performance, these psychobehavioral characteristics were not hypothesized to operate as independent determinants of qualification. Rather, they were expected to coexist with other performance-related characteristics, including training history, competitive experience, technical proficiency, physiological and neuromuscular capacity, movement efficiency, and familiarity with high-pressure competitive environments. Nutritional analyses were considered secondary and exploratory and were not hypothesized as longitudinal determinants of competitive qualification.

## 2 Materials and methods

### 2.1 Study design

This was a prospective observational study conducted during the 2025 season of the Torneio CrossFit Brasil (TCB), a national CrossFit® competition held in Brazil. The championship followed its own progressive elimination structure comprising three successive competitive stages: the online 4ALL qualifier, the in-person Super Selective events, and the in-person national Finals.

The terminology used throughout the present manuscript refers specifically to the internal competitive structure of the TCB. Thus, “qualifier”, “Super Selective”, and “Finals” should not be interpreted as stages of the official CrossFit Games qualification pathway. The present investigation evaluated athletes throughout the complete competitive cycle of the 2025 TCB season. Because progression through the championship depended on competitive qualification, the composition and number of athletes available for assessment changed across successive stages. Accordingly, analyses across competitive stages were interpreted as comparisons of the psychobehavioral and recovery-related profiles of athletes represented at each stage within an elimination-based prospective cohort and not as evidence that competitive progression itself caused psychological improvement.

The primary analytical framework comprised two complementary components. First, psychobehavioral and recovery-related profiles were examined across the successive competitive stages. Second, baseline and stage-specific characteristics were examined in relation to subsequent qualification status. Training-related variables were evaluated as contextual characteristics of the athletes, whereas anthropometric and nutritional analyses constituted a secondary exploratory component intended to characterize the cohort within the ecological competitive setting. Data collection was conducted between February and September 2025, encompassing the competitive period from the initial 4ALL qualifier to the national Finals. The study was conducted and reported according to the Strengthening the Reporting of Observational Studies in Epidemiology (STROBE) guidelines. Because competitive progression in CrossFit® is inherently multifactorial, the investigated psychobehavioral variables were interpreted as components associated with progression rather than as comprehensive or independent determinants of competitive performance.

### 2.2 Participants

A total of 239 CrossFit® athletes officially registered in the 2025 season of the Torneio CrossFit Brasil (TCB) were included in the study. Participants were distributed across the official competitive divisions established by the TCB organization, including Elite, Master, Teen, and Adapted categories. These divisions were defined according to the competition regulations and reflected age, eligibility, and category-specific competitive characteristics. Importantly, competitive division was not considered an independent or continuous measure of athletic expertise. Competitive expertise was not directly quantified through standardized performance rankings, previous championship results, technical-skill assessments, or physiological performance testing. Training experience and previous competitive experience were collected as contextual indicators of athletic background but were not considered direct measures of expertise. Accordingly, category-related findings were interpreted as differences between official competitive divisions rather than as evidence of a hierarchical expertise effect.

The primary analyses retained athletes from all eligible competitive divisions because the study was designed to characterize the real competitive population participating in the national championship rather than a laboratory-selected homogeneous subgroup. This approach was intended to preserve the ecological representativeness of the investigated competition. Nevertheless, because age, competitive demands, training background, and other characteristics may differ substantially between divisions, competitive category was explicitly evaluated in interaction models and additional sensitivity analyses were performed to assess the robustness of the principal findings.

The sample consisted of 121 men (50.6%) and 118 women (49.4%), distributed across different official competitive divisions established by the tournament organization, including elite, master, teen, and adapted categories. Participants presented a mean age of 32.59 ± 8.46 years, body mass of 76.3 ± 12.0 kg, height of 171.91 ± 8.80 cm, and body mass index (BMI) of 25.80 ± 2.55 kg/m^2^. Regarding training background, 74.9% of athletes reported training 5–6 days per week, while training and competitive experience were broadly distributed across the predefined categories. Most athletes reported typical training sessions lasting 61–120 min (77.0%), and 52.3% reported completing two training sessions per day. Detailed demographic, anthropometric, training-background, and support characteristics are presented in Table 1.

**Table 1:** Demographic, anthropometric, training-background, and support characteristics of athletes included in the study.

Variable	Total sample (*n* = 239)
Demographic and anthropometric characteristics	
Age (years)	32.59 ± 8.46
Body mass (kg)	76.3 ± 12.0
Height (cm)	171.91 ± 8.80
BMI (kg/m^2^)	25.80 ± 2.55
Sex	
Male	121 (50.6%)
Female	118 (49.4%)
Competitive division	
Elite	106 (44.4%)
Master	102 (42.7%)
Teen	17 (7.1%)
Adapted	14 (5.9%)
CrossFit® training experience	
0–1 year	32 (13.4%)
2–3 years	45 (18.8%)
4–5 years	43 (18.0%)
6–7 years	38 (15.9%)
8–9 years	38 (15.9%)
>10 years	43 (18.0%)
Competitive experience	
0–1 year	42 (17.6%)
2–3 years	44 (18.4%)
4–5 years	40 (16.7%)
6–7 years	43 (18.0%)
8–9 years	36 (15.1%)
>10 years	34 (14.2%)
Weekly training frequency	
3–4 days/week	38 (15.9%)
5–6 days/week	179 (74.9%)
7 days/week	22 (9.2%)
Typical training-session duration	
<60 min	18 (7.5%)
61–90 min	75 (31.4%)
91–120 min	109 (45.6%)
>120 min	37 (15.5%)
Daily training sessions	
1 session/day	114 (47.7%)
2 sessions/day	125 (52.3%)
Support and injury-related characteristics	
Nutritionist follow-up	142 (59.4%)
Psychological support	56 (23.4%)
Previous injury history	122 (51.0%)

Continuous variables are expressed as mean ± standard deviation (SD); categorical variables are expressed as *n* (%). BMI, body mass index. Training-related variables represent self-reported training-background characteristics and should not be interpreted as objective measures of internal or external training load.

During the competitive season, the number of athletes decreased according to the official elimination structure of the championship. A total of 239 CrossFit® athletes officially registered in the 2025 TCB season constituted the study cohort. All 239 athletes completed the initial study assessment and met the questionnaire completeness criterion. According to subsequent competitive status, 108 athletes were eliminated after the 4ALL qualifier, 92 qualified for the Super Selective stage but did not progress to the national Finals, and 39 athletes reached the Finals. Thus, 131 athletes qualified from 4ALL to the Super Selective stage. Among the 131 athletes who qualified for Super Selective, 127 completed the psychobehavioral assessment during this stage, comprising 92 athletes subsequently eliminated at Super Selective and 35 eventual finalists. Four athletes who later reached the Finals did not contribute a Super Selective assessment. All 39 finalists completed the final-stage assessment. Therefore, the phase-specific psychobehavioral samples comprised *n* = 239 at 4ALL, *n* = 127 at Super Selective, and *n* = 39 at the Finals.

Eligibility criteria were: (1) official registration as an athlete in the 2025 TCB season; (2) active participation in CrossFit® training at the time of recruitment and preparation to compete in the registered TCB division; (3) meeting the age and eligibility requirements established for the corresponding official competition division; and (4) providing valid data for at least one of the study assessment phases. Athletes under 18 years of age participated only after electronic authorization from a parent or legal guardian.

Exclusion criteria included: (1) questionnaire assessments containing one or more unanswered items; (2) duplicate responses identified during database screening; (3) inconsistent or implausible self-reported information; and (4) failure to meet the eligibility requirements of the study. Complete responses to all questionnaire items were mandatory for analytical inclusion; no partially completed questionnaire was scored, and no missing values were imputed. Participants were recruited voluntarily through online dissemination and social media platforms directed toward CrossFit® athletes. All athletes were informed about the objectives, procedures, and voluntary nature of participation prior to completing the questionnaires.

### 2.3 Data collection procedures

Participants were recruited through an online invitation disseminated via digital platforms and social media channels targeting CrossFit® athletes. Eligibility was restricted to athletes officially registered in the 2025 TCB season. All participants voluntarily agreed to participate and completed the questionnaires electronically through an online survey platform.

Data collection was performed longitudinally across three distinct phases of the championship season, corresponding to the official structure of the competition established by the tournament organization:

1. 4ALL—the initial online qualifying stage open to registered athletes;
2. Super Selective—regional in-person qualifying events;
3. National Finals—the final in-person competitive stage involving athletes who qualified through the Super Selective events.

According to the official 2025 competition structure, the 4ALL consisted of a 9-day online competition in which athletes performed standardized workouts and submitted their results through video recordings according to predefined movement standards and judging criteria. Athletes achieving qualifying positions advanced to one of four in-person Super Selective events, each conducted over 2 days. Athletes qualifying through the Super Selective stage subsequently advanced to the 4-day national Finals.

Psychobehavioral assessments were conducted within a standardized 7-day pre-competition window preceding each competitive stage. For the 4ALL qualifier, questionnaires were completed during the 7 days before the beginning of the 9-day online competition. For Super Selective, assessments were completed during the 7 days preceding the respective 2-day in-person event, whereas final-stage assessments were completed during the 7 days preceding the 4-day national Finals. The same psychobehavioral battery was applied at each stage. This procedure was designed to characterize pre-competition mood, anxiety, stress, and recovery while minimizing contamination by acute responses to competitive workouts. Questionnaires were not administered during, between, or immediately after competitive events. Although the assessment window was standardized to the 7 days preceding each stage, the exact interval between questionnaire completion and competition onset could vary within this period. Demographic and training-related information, including sex, age, competitive category, training frequency, and competitive experience, was also collected in the first contact with the assessment.

Participants were categorized according to the official competitive divisions established by the tournament organization, including categories based on sex, age group, and competitive level. Due to the elimination-based structure of the competition, the number of participants progressively decreased throughout the season as athletes advanced to subsequent phases. Completion of one competitive-phase assessment did not require completion of all subsequent assessments. Because the championship followed an elimination structure, eligibility for later assessments depended primarily on competitive qualification, while occasional non-response generated phase-specific missing observations.

Data collection occurred between February and September 2025, encompassing the entire competitive progression from the open online qualifiers to the national finals. The Figure 1 shows the main characteristics of the procedure and the phases of the data collection.

**Figure 1:**
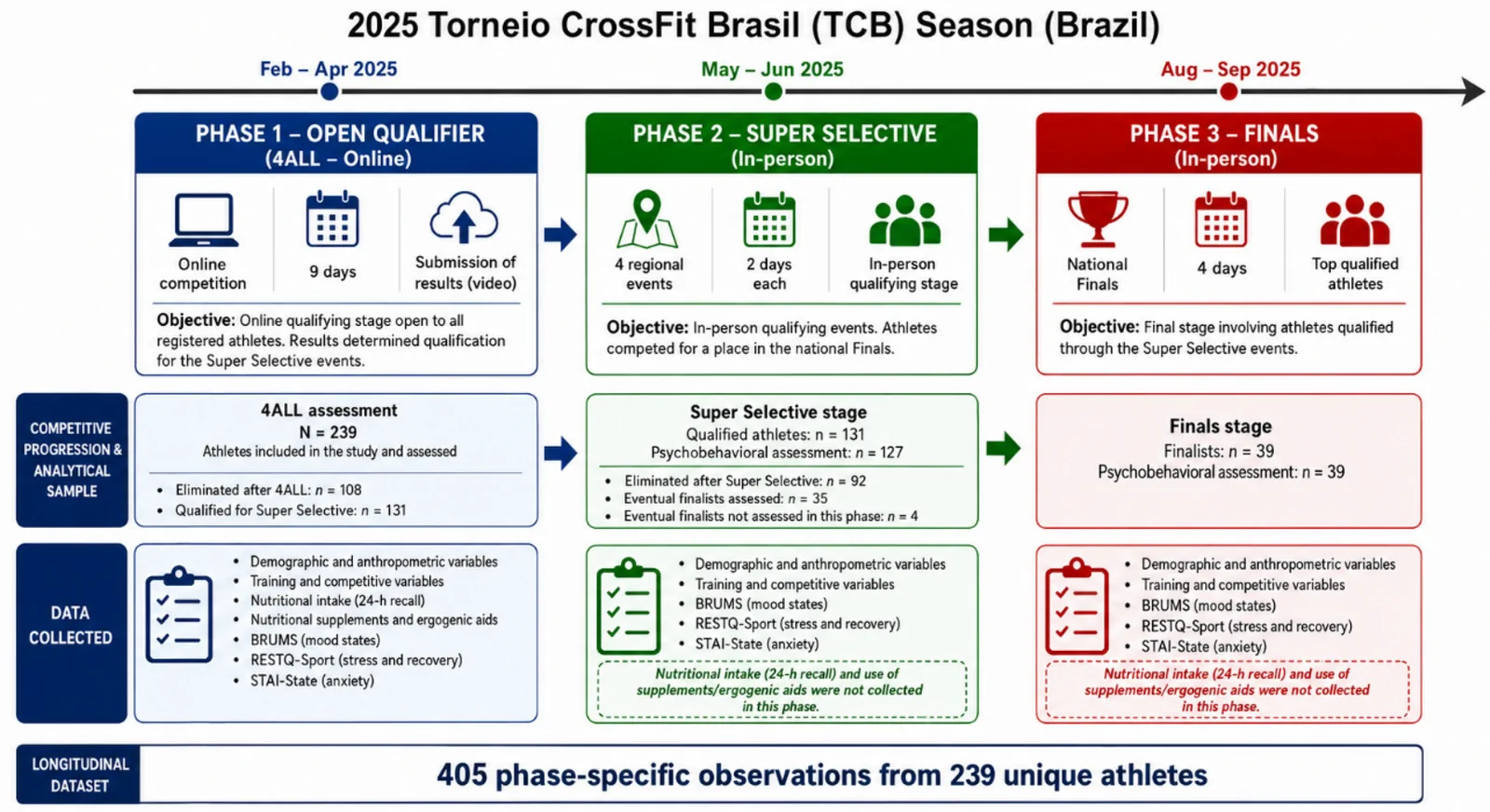
Competitive progression and phase-specific analytical sample flow throughout the 2025 torneio crossFit brasil season.

### 2.4 Psychological, training and nutritional assessments

#### 2.4.1 Mood states

Mood States were assessed using the Brunel Mood Scale (BRUMS), a psychometric instrument developed to evaluate transient mood responses in sport and exercise contexts, validated in Brazilian population by Rohlfs et al. (29). The scale consists of 24 items distributed across six dimensions: tension, depression, anger, vigor, fatigue, and confusion. Each dimension is composed of four items rated using a 5-point Likert scale ranging from 0 (“not at all”) to 4 (“extremely”), according to how participants felt at the time of assessment. The BRUMS allows the calculation of individual subscale scores and the Total Mood Disturbance (TMD) index, calculated using the following formula: TMD=(Tension+Depression+Anger+Fatigue +Confusion)−Vigor+100 (30). Higher TMD scores indicate greater overall mood disturbance, whereas higher vigor scores reflect more favorable mood-related states. The BRUMS has demonstrated adequate validity and reliability for Brazilian athletic populations and has been widely applied in sport psychology investigations involving competitive athletes.

#### 2.4.2 Stress and recovery

Stress and recovery states were evaluated using the Recovery-Stress Questionnaire for Athletes (RESTQ-Sport) (31, 32). The instrument assesses the frequency of stress-related and recovery-related experiences associated with training and competition. The version used in the present study included dimensions related to general stress, specific sport-related stress, general recovery, and sport-specific recovery. Items were answered using 7-point Likert scale ranging from 0 (“never”) to 6 (“always”), considering experiences occurring during the previous 3 days and nights. Based on the instrument structure, composite indices of general stress, specific stress, global stress, general recovery, specific recovery, and global recovery were calculated. Higher stress scores indicate greater psychophysiological overload, whereas higher recovery scores represent more favorable recovery conditions and adaptive balance between training demands and recovery capacity. The RESTQ-Sport has been validated for athletic populations and is frequently used in longitudinal monitoring of athletes exposed to high training and competitive loads.

#### 2.4.3 Competitive anxiety

Competitive anxiety was assessed using the State-Trait Anxiety Inventory—State version (STAI-State) (33). The instrument evaluates transient anxiety symptoms associated with situational emotional responses. The questionnaire consists of 20 items answered on a 4-point Likert scale ranging from 1 (“not at all”) to 4 (“very much so”). The total score ranges from 20 to 80 points, with higher scores indicating greater levels of state anxiety. The STAI has been extensively validated in sport and exercise psychology and demonstrates adequate psychometric properties for monitoring anxiety responses in competitive settings.

#### 2.4.4 Training and competitive variables

Additional training and competitive characteristics were obtained using a structured self-report questionnaire developed for the present investigation. Information included weekly training frequency, number of daily training sessions, typical session duration, CrossFit® training experience, competitive experience, and official competitive category. These variables were collected to characterize the athletes’ training background and habitual involvement in the modality. Importantly, these self-reported characteristics were not considered measures of objective training load. The study did not quantify session-specific training volume or intensity, session-RPE-derived load, heart-rate-based training load, workload accumulation, or other internal or external load metrics. Accordingly, the training variables were interpreted as contextual indicators of training background rather than quantitative measures of training stress.

#### 2.4.5 Nutritional assessment and anthropometric variables

Dietary intake was assessed using a single self-reported 24-h dietary recall administered electronically during the open qualifier phase. Participants reported all foods, beverages, dietary supplements, and ergogenic aids consumed during the previous 24 h, including meal timing and estimated portion sizes. Total energy intake and macronutrient intake were estimated as kcal/day and grams/day. Because only one 24-h recall was collected, dietary data were interpreted as an estimate of recent intake rather than habitual dietary intake. This methodological limitation is relevant because dietary recalls are susceptible to day-to-day variability and misreporting, particularly in physically active populations and individuals with higher body mass (12, 34).

Body mass (kg) and height converted to meters (m) were used to calculate body mass index (BMI), according to the following equation: $\mathrm{BMI}=\frac{\mathrm{Body}\;\mathrm{Mass}(\mathrm{kg})}{\mathrm{Heigh}t^{2}(m^{2})}$. BMI values were expressed in kg/m^2^ and used in the nutritional and regression analyses performed throughout the study.

### 2.5 Sample size and statistical power

The present investigation was conducted during an official national competitive season; participant recruitment was based on convenience sampling involving athletes officially registered in the championship. Therefore, all eligible athletes who voluntarily agreed to participate during the data collection period were included in the study.

A *post hoc* statistical power analysis was performed using G*Power software, version 3.1.9, considering the final sample size, three competitive phases, an alpha level of 0.05, and a moderate effect size (ES). The analysis indicated statistical power greater than 0.80 for detecting moderate effects in repeated-measures comparisons. However, because this was an observational study based on convenience sampling within an official competitive season, the power analysis was interpreted descriptively and not as a substitute for *a priori* sample size planning.

Given the relatively small number of finalists and the number of predictors included in the multinomial logistic regression model, these analyses were interpreted as exploratory. The ratio between the number of competitive progression events and the number of predictors was considered when interpreting model stability and the risk of overfitting. The progressive reduction in sample size throughout the season reflected the elimination-based structure of the competition rather than participant dropout, thereby representing an inherent characteristic of the competitive model investigated in the present study.

### 2.6 Statistical analysis

Descriptive statistics were initially calculated for all study variables. Continuous variables are presented as mean ± standard deviation (SD) when approximately normally distributed or as median [interquartile range] when distributional assumptions were not satisfied. Categorical variables are presented as absolute and relative frequencies. Distributional characteristics were evaluated using the Shapiro–Wilk test in conjunction with visual inspection of the data distributions. No missing psychobehavioral values were imputed. Analyses were performed using the complete observations available for each analytical component, and the corresponding analytical sample size is reported where appropriate.

The primary analysis examined stage-related differences in psychobehavioral and recovery-related outcomes using linear mixed models (LMMs). Competitive stage was entered as a three-level fixed effect (4ALL, Super Selective, and Finals), and participant identification was included as a random intercept to account for within-participant dependency arising from repeated observations. The mixed-model approach incorporated all available complete phase-specific assessments and therefore accommodated the unbalanced repeated-measures structure generated primarily by progressive competitive elimination and, to a lesser extent, by occasional non-response at subsequent stages. Fixed effects were evaluated using likelihood-ratio *χ*^2^ tests, and stage contrasts were reported as fixed-effect coefficients (*β*), 95% confidence intervals (CI), and *p*-values. Marginal and conditional *R*^2^ values were calculated according to Nakagawa's method to quantify the variance explained by the fixed effects and by the complete mixed model, respectively. Because the composition of the athlete sample changed systematically across stages, these models were interpreted as describing stage-related psychobehavioral profiles rather than as direct evidence of within-athlete improvement over time.

To investigate whether stage-related patterns differed according to athlete characteristics, additional LMMs examined competitive stage × sex and competitive stage × competitive division interactions. Interaction coefficients (*β*), 95% CI, and *p*-values were reported. Because Teen and Adapted athletes were represented by relatively small numbers at later competitive stages, sensitivity analyses were conducted to assess whether the principal findings were driven by the heterogeneity of the pooled competitive divisions. The primary LMMs for Total Mood Disturbance (TMD), state anxiety, Global Stress, and Global Recovery were first repeated after restricting the sample to Elite and Master athletes. Complementary models were then performed separately for Elite and Master athletes. Teen and Adapted athletes were not modeled separately because the small number reaching the Finals did not permit sufficiently stable division-specific estimates.

Competitive progression was subsequently examined using the pre-competition assessment obtained before the initial 4ALL stage. Athletes were classified into three mutually exclusive groups according to the furthest competitive stage reached during the championship: eliminated after 4ALL (*n* = 108), eliminated after Super Selective (*n* = 92), and finalists (*n* = 39). Because baseline psychobehavioral variables did not consistently satisfy parametric assumptions, between-group comparisons were performed using Kruskal–Wallis tests. Results are presented as median [interquartile range], Kruskal–Wallis *H* statistic, *p*-value, and epsilon squared (*ε*^2^) as a non-parametric effect-size estimate. A multinomial logistic regression model was additionally used to explore associations between baseline characteristics and subsequent competitive progression, with athletes eliminated after 4ALL serving as the reference outcome category. Demographic, anthropometric, psychobehavioral, and recovery-related variables included in the adjusted model are reported in Table 2. Regression coefficients (*β*), odds ratios (OR), 95% CI, and *p*-values were reported. Given the relatively small number of finalists in relation to the number of covariates, the multinomial model was considered exploratory and was not interpreted as establishing independent causal determinants of qualification.

**Table 2:** Multinomial logistic regression model for factors associated with competitive progression.

Predictor	Eliminated after super selective vs. Eliminated after 4ALL				Finalists’ vs. eliminated after 4ALL			
	*β*	OR	95% CI	*p*-value	*β*	OR	95% CI	*p*-value
Age	0.083	1.086	0.942–1.253	0.254	0.040	1.041	0.835–1.297	0.72
BMI	−0.004	0.996	0.885–1.121	0.949	−0.031	0.969	0.820–1.146	0.71
Performance pressure	−0.092	0.912	0.619–1.344	0.641	−0.064	0.938	0.547–1.609	0.81
Sleep recovery quality	0.220	1.246	0.785–1.979	0.350	0.336	1.400	0.751–2.607	0.29
Anxiety	−0.095	0.909	0.874–0.946	<0.001**	−0.134	0.874	0.831–0.919	<0.01*
TMD	−0.405	0.667	0.572–0.779	<0.001**	−0.639	0.528	0.436–0.640	<0.01*
Global Stress	−0.679	0.451	0.283–0.813	<0.01*	−0.946	0.413	0.183–0.79	<0.01*
Global Recovery	0.621	1.881	1.264–3.264	<0.001**	0.956	2.612	1.413–4.877	<0.001**
Vigor	0.070	1.073	0.875–1.315	0.499	0.148	1.159	0.871–1.544	0.31
Male sex	−0.541	0.582	0.201–1.690	0.320	0.034	1.034	0.227–4.712	0.96
Elite category	−0.456	0.634	0.063–6.370	0.699	1.462	4.314	0.182–102.322	0.36
Master 35–39 category	−0.705	0.494	0.043–5.631	0.570	1.166	3.211	0.091–113.299	0.52
Master 40–44 category	−2.750	0.064	0.003–1.205	0.066	0.321	1.378	0.012–154.775	0.89
Master 45–49 category	−3.144	0.043	0.001–1.383	0.076	−0.110	0.895	0.005–173.208	0.96
Teen category	1.568	4.795	0.143–161.128	0.382	1.547	4.695	0.029–763.310	0.55

OR, odds ratio; CI, confidence interval; BMI, body mass index; TMD, Total Mood Disturbance. Multinomial logistic regression models were adjusted to explore factors associated with competitive progression. Reference outcome category: athletes eliminated after 4ALL.

* *p* < 0.05.

** *p* < 0.001.

To specifically distinguish between competitive selection and within-athlete variation, two complementary analyses were conducted. First, athletes facing the same competitive stage were compared according to their subsequent qualification status. At 4ALL, athletes who were subsequently eliminated (*n* = 108) were compared with those who qualified for Super Selective (*n* = 131). At Super Selective, athletes subsequently eliminated (*n* = 92) were compared with the assessed athletes who subsequently qualified for the Finals (*n* = 35). These comparisons are temporally relevant because psychobehavioral assessments were completed within the 7 days preceding the respective competitive stage and therefore preceded the subsequent qualification outcome. Between-group comparisons were performed using Mann–Whitney *U* tests, with Cliff's delta (*δ*) reported as the effect-size estimate. Because 14 psychobehavioral outcomes were examined within each stage, *p*-values were adjusted separately within each stage using the Benjamini–Hochberg false-discovery-rate (FDR) procedure.

Second, a complete-case sensitivity analysis was conducted among the 35 finalists who completed psychobehavioral assessments before all three competitive stages. Restricting this analysis to the same athletes across 4ALL, Super Selective, and Finals held sample composition constant and therefore removed progressive between-stage selection as an explanation for the observed differences. Overall, within-athlete stage effects were examined using Friedman tests. When a significant overall effect was identified, pairwise comparisons were performed using Wilcoxon signed-rank tests, with Benjamini–Hochberg FDR correction applied to the three pairwise stage comparisons within each outcome. These analyses were interpreted as evidence of within-athlete stage-related variation rather than as causal effects of competitive progression.

Training-background variables were examined separately as contextual characteristics potentially associated with competitive progression. Because training experience, competitive experience, weekly training frequency, typical session duration, and number of daily training sessions were recorded as categorical or ordinal variables, their distributions across competitive-progression groups were initially compared using chi-square tests, with Cramer's *V* reported as the measure of association. Complementary Kruskal–Wallis analyses were performed for ordinal representations of these variables where appropriate. Relationships between training-background characteristics and the principal psychobehavioral outcomes (TMD, state anxiety, Global Stress, and Global Recovery) were explored using Spearman rank-order correlations (*ρ*). *P*-values arising from these complementary training analyses were adjusted using the Benjamini–Hochberg FDR procedure. These variables were interpreted as indicators of training background rather than measures of internal or external training load.

Nutritional and anthropometric analyses were conducted in the subsample of athletes with available dietary data. Total energy intake and carbohydrate, protein, and fat intake were summarized descriptively. To explore the association between macronutrient intake and BMI, a multiple linear regression model was fitted with BMI as the dependent variable and carbohydrate, protein, and fat intake (g/day) as independent variables. Total energy intake was not simultaneously entered into the regression model because it is mathematically derived from macronutrient intake and its simultaneous inclusion produced substantial multicollinearity. Model fit was evaluated using *R*^2^, adjusted *R*^2^, and the overall *F* statistic. Regression diagnostics included assessment of residual autocorrelation using the Durbin–Watson statistic, residual normality using the Shapiro–Wilk test, and multicollinearity using variance inflation factors (VIF) and tolerance values. Nutritional analyses were considered secondary and exploratory because dietary intake was derived from a single 24-h dietary recall.

Statistical significance was established at *p* < 0.05 for primary analyses. For analyses involving multiple simultaneous comparisons, statistical inference was based on Benjamini–Hochberg FDR-adjusted *p*-values as specified above. All statistical tests were two-sided. Analyses were performed using Jamovi statistical software, version 2.6.19 (The Jamovi Project, Sydney, Australia).

## 3 Results

The overall cohort comprised 239 athletes. Following the 4ALL qualifier, 108 athletes were eliminated and 131 qualified for the Super Selective stage. Of these 131 athletes, 92 did not subsequently progress beyond the Super Selective stage, whereas 39 reached the national Finals. Complete psychobehavioral data were available for all 239 athletes during the 4ALL assessment, comprising 108 athletes subsequently eliminated after 4ALL, 92 subsequently eliminated after Super Selective, and 39 eventual finalists. At the Super Selective stage, psychobehavioral data were available for 127 athletes, including 92 athletes subsequently eliminated and 35 eventual finalists. All 39 finalists contributed data during the national Finals. Accordingly, the longitudinal mixed-model dataset comprised 405 phase-specific observations from 239 unique athletes.

### 3.1 Psychobehavior profiles across competitive stages

Significant differences in mood state variables were observed across competitive progression phases (Table 3). Athletes who remained in the competition until the Super Selective and Final stages presented lower scores for negative mood dimensions, including tension, depression, fatigue, confusion, and TMD, whereas higher vigor scores were observed among athletes who reached later stages. The largest effect was identified for TMD (*χ*^2^ = 184.311; marginal *R*^2^ = 0.501), indicating marked differences in overall mood disturbance according to competitive progression status. Significant differences were also identified for tension (*χ*^2^ = 73.185), fatigue (*χ*^2^ = 45.821), and confusion (*χ*^2^ = 47.864). Although smaller in magnitude, anger also demonstrated statistically significant differences across competitive phases.

**Table 3:** Psychobehavioral profiles across competitive stages according to linear mixed-model analysis.

Variable	4ALL	Super Selective	Finals	Fixed Effect (*χ*^2^)	Comparison	*β*	95% CI	*p*-value	Marginal *R*^2^	Conditional *R*^2^
Tension	7.68 ± 2.83	6.41 ± 2.78	5.48 ± 2.55	73.185	Super Selective vs. 4ALL	−0.668	−0.899 to −0.438	<0.01**	0.465	0.939
					Finals vs. 4ALL	−1.500	−1.882 to −1.119			
Depression	8.01 ± 2.88	6.63 ± 2.80	5.92 ± 3.17	47.700	Super Selective vs. 4ALL	−0.876	−1.144 to −0.608	<0.01**	0.450	0.918
					Finals vs. 4ALL	−0.965	−1.409 to −0.520			
Anger	7.98 ± 2.90	7.51 ± 3.15	7.64 ± 3.45	11.712	Super Selective vs. 4ALL	−0.531	−0.859 to −0.203	0.01**	0.421	0.875
					Finals vs. 4ALL	−0.575	−1.119 to −0.032			
Vigor	7.77 ± 3.01	8.77 ± 3.14	9.71 ± 3.53	18.053	Super Selective vs. 4ALL	0.541	0.284 to 0.799	<0.01**	0.468	0.934
					Finals vs. 4ALL	0.467	0.040 to 0.894			
Fatigue	7.94 ± 2.88	6.41 ± 2.84	5.48 ± 2.30	45.821	Super Selective vs. 4ALL	−0.552	−0.777 to −0.326	<0.01**	0.466	0.946
					Finals vs. 4ALL	−1.126	−1.498 to −0.754			
Confusion	8.15 ± 2.80	7.65 ± 2.98	6.64 ± 2.87	47.864	Super Selective vs. 4ALL	−0.616	−0.825 to −0.408	<0.01**	0.477	0.951
					Finals vs. 4ALL	−0.916	−1.260 to −0.571			
TMD	132.01 ± 7.33	125.82 ± 7.03	121.53 ± 7.55	184.311	Super Selective vs. 4ALL	−3.859	−4.514 to −3.204	<0.01**	0.501	0.931
					Finals vs. 4ALL	−5.608	−6.692 to −4.524			

BRUMS, Brunel Mood Scale; TMD, Total Mood Disturbance; LMM, Linear Mixed Model; *β*, fixed effect coefficient estimated by linear mixed model; CI, confidence interval; *R*^2^, coefficient of determination. Effect sizes are expressed as marginal *R*^2^. Standardized *β* values were estimated by dividing regression coefficients by pooled standard deviation of each outcome. Marginal and conditional *R*^2^ values were calculated according to Nakagawa's method.

* *p* < 0.05.

** *p* < 0.001.

Similarly, athletes advancing to later competitive stages demonstrated lower stress-related scores and higher recovery-related scores throughout the competitive season (Table 4). General stress, specific stress, and global stress were lower among athletes progressing to Super Selective and Final stages, whereas general recovery, specific recovery, and global recovery scores were progressively higher in these groups. The strongest recovery-related effects were identified for global recovery (*χ*^2^ = 84.833; marginal *R*^2^ = 0.481) and general recovery (*χ*^2^ = 58.930; marginal *R*^2^ = 0.468). Collectively, these findings suggest that athletes reaching later competitive stages presented more favorable psychobehavioral, stress-related, and recovery-related profiles throughout the season.

**Table 4:** Stress and recovery profiles across competitive stages according to linear mixed-model analysis.

Variable	4ALL	Super Selective	Finals	Fixed Effect (χ^2^)	Comparison	*β*	95% CI	*p*-value	Marginal *R*^2^	Conditional *R*^2^
General Stress	2.01 ± 0.25	1.90 ± 0.27	1.90 ± 0.31	65.621	Super Selective vs. 4ALL	−0.108	−0.141 to −0.075	<0.01**	0.435	0.879
					Finals vs. 4ALL	−0.133	−0.168 to −0.058			
Specific Stress	2.06 ± 0.38	1.90 ± 0.39	1.94 ± 0.38	58.431	Super Selective vs. 4ALL	−0.152	−0.199 to −0.105	<0.01**	0.419	0.904
					Finals vs. 4ALL	−0.118	−0.196 to −0.040			
Global Stress	2.04 ± 0.23	1.90 ± 0.22	1.92 ± 0.23	119.419	Super Selective vs. 4ALL	−0.141	−0.170 to −0.112	<0.01**	0.461	0.916
					Finals vs. 4ALL	−0.124	−0.173 to −0.075			
General Recovery	2.03 ± 0.31	2.22 ± 0.32	2.37 ± 0.30	58.93	Super Selective vs. 4ALL	0.192	0.154 to 0.231	<0.01**	0.468	0.932
					Finals vs. 4ALL	0.341	0.278 to 0.404			
Specific Recovery	1.98 ± 0.41	2.19 ± 0.39	2.47 ± 0.28	76.073	Super Selective vs. 4ALL	0.213	0.164 to 0.262	<0.01**	0.428	0.898
					Finals vs. 4ALL	0.487	0.406 to 0.568			
Global Recovery	2.01 ± 0.26	2.21 ± 0.22	2.42 ± 0.21	84.833	Super Selective vs. 4ALL	0.128	0.099 to 0.157	<0.01**	0.481	0.956
					Finals vs. 4ALL	0.339	0.291 to 0.387			

LMM, Linear Mixed Model; *β*, fixed effect coefficient estimated by linear mixed model; CI, confidence interval; *R*^2^, coefficient of determination. Effect sizes are expressed as marginal *R*^2^. Standardized *β* values were estimated by dividing regression coefficients by pooled standard deviation of each outcome. Marginal and conditional *R*^2^ values were calculated according to Nakagawa's method.

* *p* < 0.05.

** *p* < 0.001.

Lower pre-competition state anxiety scores were observed among athletes represented at later competitive stages, as illustrated in Figure 2. Overall, the findings indicate that competitive progression throughout the season was associated with more favorable psychological, stress-related, and recovery-related profiles.

**Figure 2:**
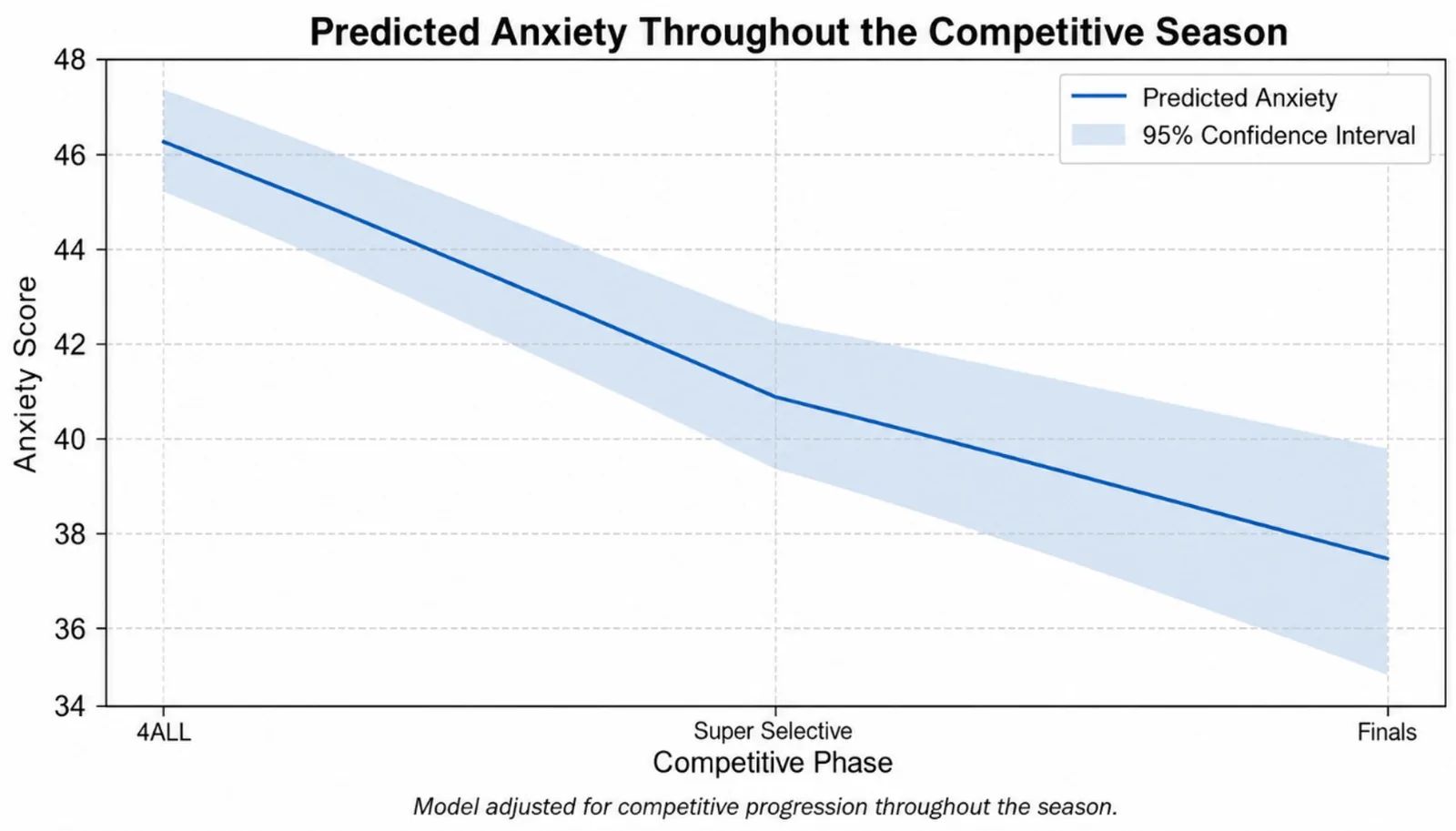
Pre-competition state anxiety across competitive stages according to linear mixed-model analysis. Assessments were conducted within the 7 days preceding each competitive stage. Values are presented as estimated marginal means ± 95% confidence intervals.

### 3.2 Interaction effects according to sex and competitive category

Interaction analyses demonstrated limited sex-related effects on psychobehavioral profiles across competitive progression phases (Table 5). Among the evaluated variables, only anger showed a statistically significant interaction between competitive phase and sex (*β* = 0.484; 95% CI: 0.017–0.951; *p* = 0.04), suggesting that this specific mood dimension may differ between men and women across the competitive stages. No significant sex-related interactions were observed for tension, depression, vigor, confusion, TMD, stress-related variables, recovery-related variables, or anxiety. In contrast, the competitive category showed significant interaction effects for several psychobehavioral variables, including tension, anger, vigor, confusion, TMD, general stress, specific stress, global stress, general recovery, and specific recovery. The largest interaction effects were observed for anger (*β* = −1.567; *p* < 0.001) and TMD (*β* = −3.468; *p* < 0.001), indicating that psychobehavioral profiles differed according to competitive category. No significant category-related interactions were observed for depression, global recovery, or anxiety.

**Table 5:** Interaction effects according to LMM analysis.

Variable	Competitive phase and sex			Competitive phase and category		
	*β*	95% CI	*p*-value	*β*	95% CI	*p*-value
Tension	−0.181	−0.509 to 0.147	0.28	−0.916	−1.690 to −0.142	0.01*
Depression	0.153	−0.234 to 0.540	0.44	−0.643	−1.373 to 0.087	0.08
Anger	0.484	0.017 to 0.951	0.04*	−1.567	−2.402 to −0.732	<0.01**
Vigor	0.227	−0.144 to 0.598	0.23	0.983	0.271 to 1.695	0.01*
Confusion	−0.172	−0.473 to 0.129	0.26	−1.005	−1.757 to −0.253	0.01*
TMD	−0.638	−1.584 to 0.308	0.18	−3.468	−5.091 to −1.845	<0.01**
General Stress	0.045	−0.008 to 0.098	0.09	0.163	0.061 to 0.265	0.01*
Specific Stress	0.032	−0.018 to 0.082	0.21	0.108	0.001 to 0.215	0.04*
Global Stress	0.038	−0.010 to 0.086	0.11	0.136	0.064 to 0.208	<0.01**
General Recovery	−0.041	−0.088 to 0.006	0.08	0.191	0.102 to 0.280	<0.01**
Specific Recovery	−0.029	−0.073 to 0.015	0.19	−0.177	−0.264 to −0.090	<0.01**
Global Recovery	−0.036	−0.080 to 0.008	0.10	0.066	−0.031 to 0.163	0.18
Anxiety	−1.823	−3.825 to 0.179	0.07	−5.076	−14.691 to 4.539	0.30

LMM, Linear Mixed Model; *β*, interaction coefficient estimated by linear mixed model; CI, confidence interval. Significant interaction terms indicate that stage-related psychobehavioral patterns differed according to sex or competitive category.

* *p* < 0.05.

** *p* < 0.001.

Given the unequal representation of competitive divisions across successive stages, additional sensitivity analyses were conducted. After restricting the analysis to Elite and Master athletes, the principal stage-related patterns remained statistically significant for TMD, state anxiety, global stress, and global recovery. The direction of these effects was also preserved when Elite and Master athletes were examined separately. Within the Elite division, later competitive stages remained associated with lower TMD, lower anxiety, lower global stress, and higher global recovery. A comparable pattern was observed among Master athletes. These findings indicate that the principal psychobehavioral and recovery-related patterns identified in the pooled analyses were not solely attributable to the inclusion of Teen and Adapted athletes. However, because only one Teen athlete and three Adapted athletes reached the final assessment, division-specific inferential analyses were not considered sufficiently stable for these groups. Detailed results of these sensitivity analyses are presented in Supplementary Table S1.

Overall, these findings suggest that the competitive category may be more strongly associated with psychobehavioral differences across competitive stages than biological sex. However, these results should be interpreted cautiously because some categories, particularly teenagers and adapted divisions, included relatively small numbers of athletes.

### 3.3 Within-stage psychobehavioral differences according to subsequent qualification

To further distinguish competitive selection from stage-related differences, athletes were compared within the same competitive stage according to their subsequent qualification status. Because all assessments were completed within the 7 days preceding each respective competitive stage, psychobehavioral measures preceded the subsequent qualification outcome. Complete within-stage comparisons, including effect sizes and FDR-adjusted *p*-values, are presented in Supplementary Table S2.

During the 4ALL stage, athletes who subsequently qualified for Super Selective (*n* = 131) already displayed a more favorable psychobehavioral profile than athletes subsequently eliminated (*n* = 108). After FDR correction, qualified athletes presented lower tension, depression, fatigue, TMD, and state anxiety, together with higher vigor, General Recovery, Specific Recovery, and Global Recovery. Global Recovery showed the largest between-group effect (*δ* = 0.636), followed by fatigue (*δ* = −0.465), General Recovery (*δ* = 0.445), TMD (*δ* = −0.441), and Specific Recovery (*δ* = 0.420). Global Stress showed only a nominal between-group difference and did not remain statistically significant after correction for multiple comparisons. A more selective pattern emerged during Super Selective.

Among athletes assessed during this stage, those who subsequently reached the Finals (*n* = 35) showed higher General Recovery, Specific Recovery, and Global Recovery than athletes subsequently eliminated (*n* = 92), with these differences remaining significant after FDR correction. The strongest effect was observed for Global Recovery (*δ* = 0.504), followed by Specific Recovery (*δ* = 0.349) and General Recovery (*δ* = 0.297). Although eventual finalists showed nominally higher vigor and lower TMD, these differences did not remain statistically significant after correction. State anxiety did not significantly differ according to subsequent finalist status. Collectively, these results indicate that broad psychobehavioral differences were already evident before qualification from the initial 4ALL stage, whereas within the more homogeneous Super Selective context, perceived recovery was the most consistent psychobehavioral characteristic associated with subsequent progression.

To evaluate whether stage-related differences could be attributed exclusively to progressive sample selection, a sensitivity analysis was performed among the 35 finalists who completed assessments at all three competitive stages, the detailed results of the complete-case within-athlete analysis and FDR-adjusted pairwise comparisons are presented in Supplementary Supplementary Table S3. Significant within-athlete stage effects were observed for TMD (Friedman *χ*^2^ = 37.12, *p* < 0.001), state anxiety (*χ*^2^ = 29.83, *p* < 0.001), Global Stress (*χ*^2^ = 31.26, *p* < 0.001), and Global Recovery (*χ*^2^ = 28.34, *p* < 0.001).

Median TMD decreased from 126 at 4ALL to 123 at Super Selective and 123 at the Finals, while state anxiety decreased from 36 to 34 and 26, respectively. Global Stress decreased from 1.99 to 1.89 and 1.88, whereas Global Recovery increased from 2.31 to 2.33 and 2.45. Pairwise analyses with false-discovery-rate correction confirmed significant stage-related differences for most comparisons, although the Super Selective-to-Finals difference in Global Stress was not significant. These findings demonstrate that the stage-related patterns observed in the complete cohort were not entirely explained by progressive selection, as within-athlete variation remained detectable when sample composition was held constant.

### 3.4 Baseline psychobehavioral characteristics associated with subsequent competitive progression

Baseline psychological characteristics differed according to competitive progression status (Table 6). Athletes advancing to later competitive phases demonstrated more favorable baseline psychological profiles, characterized by lower tension, depression, fatigue, anxiety, and total mood disturbance scores, together with higher vigor and recovery-related scores. In contrast, anger, confusion, general stress, specific stress, and global stress did not differ significantly between competitive progression groups. The largest ES were observed for global recovery (*ε*^2^ = 0.367), TMD (*ε*^2^ = 0.185), general recovery (*ε*^2^ = 0.176), fatigue (*ε*^2^ = 0.163), specific recovery (*ε*^2^ = 0.161), and anxiety (*ε*^2^ = 0.100). These findings indicate that recovery-related variables, global mood disturbance, fatigue, and anxiety showed the largest baseline differences according to subsequent competitive progression status.

**Table 6:** Baseline psychobehavioral characteristics according to subsequent competitive status.

Variable	Eliminated after 4ALL (*n* = 108)	Eliminated after super selective (*n* = 92)	Finalists (*n* = 39)	*H*	*p*-value	Effect Size (*ε*^2^)
Tension	9.00 [7.00–10.00]	7.00 [5.00–9.00]	7.00 [5.00–9.00]	16.871	<0.001**	0.063
Depression	9.00 [7.00–11.00]	7.00 [6.00–10.00]	7.00 [4.00–8.50]	19.032	<0.001**	0.072
Anger	8.00 [6.00–10.00]	8.00 [6.00–10.25]	8.00 [6.00–10.00]	0.632	0.729	<0.001
Vigor	7.00 [5.00–9.00]	8.00 [6.00–10.00]	10.00 [7.00–12.00]	17.334	<0.001**	0.065
Fatigue	9.00 [7.00–11.00]	7.00 [5.00–9.00]	6.00 [5.00–8.50]	40.482	<0.001**	0.163
Confusion	8.00 [6.00–10.00]	9.00 [7.00–11.00]	7.00 [5.00–9.50]	5.841	0.054	0.016
TMD	136.00 [131.00–139.25]	133.00 [125.75–135.00]	126.00 [120.00–132.00]	45.612	<0.001**	0.185
General Stress	2.04 [1.86–2.18]	2.00 [1.82–2.21]	1.96 [1.82–2.18]	1.035	0.596	<0.001
Specific Stress	2.08 [1.83–2.33]	2.00 [1.67–2.33]	2.08 [1.79–2.29]	3.309	0.191	0.006
Global Stress	2.07 [1.93–2.23]	2.02 [1.88–2.15]	2.00 [1.81–2.22]	4.310	0.116	0.010
General Recovery	1.90 [1.70–2.10]	2.05 [1.90–2.20]	2.25 [2.10–2.45]	43.638	<0.001**	0.176
Specific Recovery	1.81 [1.50–2.12]	2.06 [1.88–2.25]	2.25 [2.06–2.44]	40.063	<0.001**	0.161
Global Recovery	1.86 [1.74–1.99]	2.05 [1.93–2.16]	2.31 [2.19–2.39]	88.721	<0.001**	0.367
Anxiety	62.50 [42.75–70.00]	47.50 [36.75–56.75]	37.00 [28.50–56.00]	25.490	<0.001**	0.100

Values are expressed as median [interquartile range]. Between-group comparisons were performed using the Kruskal–Wallis test. *H* = Kruskal–Wallis test statistic; *ε*^2^, epsilon squared effect size; TMD, Total Mood Disturbance. Negative *ε*^2^ values were truncated to <0.001 for interpretability. Statistical significance was set at *p* < 0.05. Competitive-status groups are mutually exclusive and represent the furthest competitive stage reached by each athlete during the 2025 TCB season. All 239 athletes provided complete baseline psychobehavioral assessments.

** *p* < 0.001.

Multinomial logistic regression analyses further demonstrated that lower anxiety, lower TMD, lower global stress, and higher global recovery were associated with greater odds of advancing throughout the competitive season (Table 2). Specifically, higher global recovery scores were positively associated with progression to later competitive stages, whereas higher anxiety, TMD, and global stress scores were associated with reduced odds of advancement. Variables related to sex and competitive category did not demonstrate consistent associations with competitive progression in the adjusted models. These associations should not be interpreted as evidence that the identified variables independently determined qualification, because additional performance-related characteristics were not comprehensively assessed.

### 3.5 Training background and competitive progression

The available self-reported training-background characteristics showed no consistent differences according to competitive progression status. Training experience (*p* = 0.708; Cramer's *V* = 0.123), competitive experience (*p* = 0.188; *V* = 0.169), weekly training frequency (*p* = 0.130; *V* = 0.122), session duration (*p* = 0.228; *V* = 0.131), and number of daily training sessions (*p* = 0.115; *V* = 0.134) did not significantly differ across competitive-status groups. Complementary ordinal analyses identified nominal differences for competitive experience and weekly training frequency; however, these associations did not remain statistically significant after false-discovery-rate correction. Associations between training-background variables and the principal psychobehavioral outcomes were also generally weak and none remained significant after correction for multiple comparisons. Collectively, the available self-reported training-background variables did not consistently differentiate competitive progression groups or explain the observed psychobehavioral profiles. Detailed results of the training-background comparisons and their associations with the principal psychobehavioral outcomes are presented in Supplementary Table S4.

### 3.6 Exploratory nutritional and anthropometric analysis

Descriptive characteristics of anthropometric and dietary variables are presented in Table 7. Participants presented a mean body weight of 76.3 ± 12.0 kg, a mean height of 1.71 ± 0.08 m, and a mean BMI of 25.8 ± 2.55 kg/m^2^. Median energy intake was 1235 kcal/day, while median carbohydrate, protein, and fat intakes were 114 g/day, 93 g/day, and 32 g/day, respectively.

**Table 7:** Descriptive characteristics of anthropometric and dietary variables.

Variable	Mean ± SD	Median	Minimum	Maximum
Weight (kg)	76.3 ± 12.0	78.5	58.0	99.0
Height (m)	1.71 ± 0.08	1.70	1.56	1.94
BMI (kg/m^2^)	25.8 ± 2.55	26.0	21.0	31.0
Kcal/day	1,546 ± 599	1,235	1,200	4,290
Carbohydrates (g)	139 ± 73.2	114	80	353
Proteins (g)	116 ± 61.3	93.0	50	300
Fat (g)	40.7 ± 24.0	32.0	25	180

**Table 8:** Exploratory multiple linear regression analysis of the association between macronutrient intake and body mass index (BMI).

Predictor	*β*	*p*
Carbohydrate (g/day)	−0.001	0.877
Protein (g/day)	0.008	0.276
Fat (g/day)	0.017	0.490

Model fit: *R*^2^ = 0.049; adjusted *R*^2^ = 0.003; *F*(3,62) = 1.08; *p* = 0.366. Multicollinearity was low, with VIF values ranging from 1.38 to 1.68 across predictors.

The multiple linear regression model examined the exploratory association between macronutrient intake and BMI (Table 8). The overall model demonstrated low explanatory capacity and was not statistically significant [*R*^2^ = 0.049; adjusted *R*^2^ = 0.003; *F*(3,62) = 1.08; *p* = 0.366]. None of the individual macronutrients was significantly associated with BMI: carbohydrate intake (*β* = −0.001; *p* = 0.877), protein intake (*β* = 0.008; *p* = 0.276), and fat intake (*β* = 0.017; *p* = 0.490). These findings indicate that macronutrient intake estimated from the single 24-h dietary recall explained little of the variation in BMI within this subsample.

Graphical representations of the exploratory associations between macronutrient intake and predicted BMI are presented in Figure 3. Panels A, B, and C illustrate the associations of carbohydrate, protein, and fat intake with predicted BMI, respectively. Consistent with the regression analysis, none of the individual macronutrients showed a statistically significant association with BMI. Regression diagnostic analyses indicated adequate model assumptions. Residuals were normally distributed according to the Shapiro–Wilk test (*p* = 0.235), and no evidence of residual autocorrelation was observed based on the Durbin–Watson statistic (DW = 1.91; *p* = 0.666). Multicollinearity was low in the revised model, with VIF values ranging from 1.38 to 1.68.

**Figure 3:**
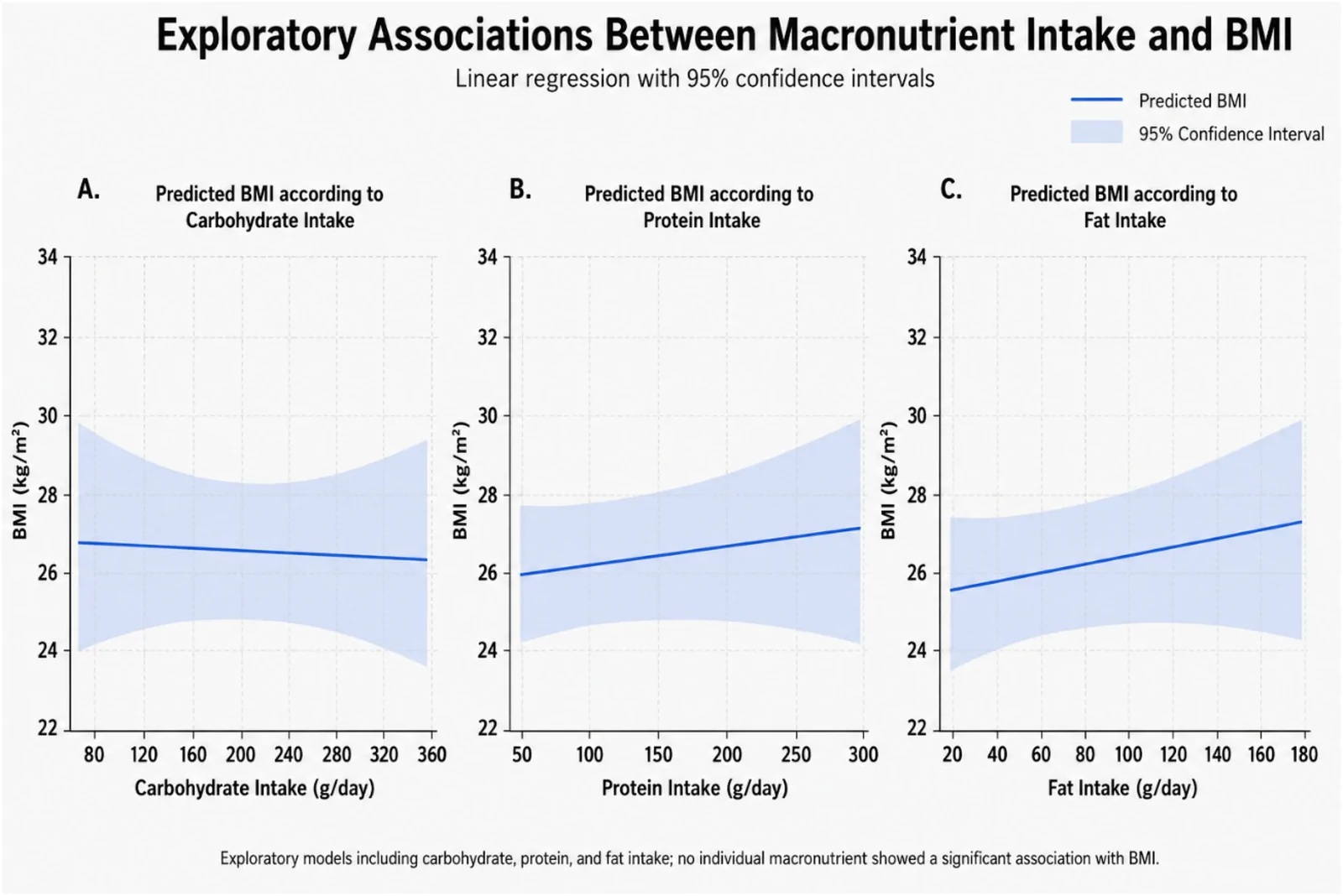
Exploratory associations between macronutrient intake and body mass index (BMI). Figure presents the exploratory associations between carbohydrate intake **(A)**, protein intake **(B)**, and fat intake **(C)** with body mass index (BMI). Solid lines represent predicted BMI values derived from the multiple linear regression model, and shaded areas represent the corresponding 95% confidence intervals. The overall model was not statistically significant [*R*^2^ = 0.049; adjusted *R*^2^ = 0.003; *F*(3,62) = 1.08; *p* = 0.366], and no individual macronutrient was significantly associated with BMI. Carbohydrate, protein, and fat intakes were expressed in g/day. BMI, body mass index.

## 4 Discussion

### 4.1 Psychobehavioral profiles across competitive stages

The principal finding of the present study was that athletes represented at later stages of the national CrossFit® competition displayed more favorable psychobehavioral and recovery-related profiles, characterized by lower anxiety, lower mood disturbance and perceived stress, and higher perceived recovery. Importantly, these findings should not be interpreted as evidence that competitive progression itself produced psychological improvement or that the identified psychobehavioral characteristics independently determined qualification. Because the championship followed a progressive elimination structure, athletes assessed at later stages represented an increasingly selected subgroup of the original cohort. Furthermore, because all psychobehavioral assessments were conducted during the 7 days preceding each competitive stage, the observed differences primarily characterize athletes’ pre-competition psychological and recovery-related status, rather than acute responses generated during or immediately after competitive workouts. This temporal design reduces the likelihood that the observed profiles merely reflect acute post-workout fatigue, emotional responses to recently completed events, or between-event fluctuations during multi-day competitions.

The complementary within-stage analyses provide important insight into the extent to which these differences reflect competitive selection. During the initial 4ALL stage, athletes who subsequently qualified already exhibited lower mood disturbance, fatigue, and anxiety and higher perceived recovery before the qualification outcome occurred. Thus, a substantial component of the more favorable profiles observed at later stages appears to reflect the selective progression of athletes who entered the competition with different pre-competition psychobehavioral characteristics. However, the pattern became more specific as the competitive field became increasingly selective. Among athletes facing the same Super Selective context, perceived recovery, particularly Global Recovery, was the most consistent characteristic differentiating athletes who subsequently reached the Finals from those who were eliminated. In contrast, anxiety and TMD did not remain significantly different after correction for multiple comparisons. These findings suggest that the characteristics differentiating athletes during the broad initial qualifier may not necessarily be the same as those distinguishing athletes within a progressively more homogeneous competitive field.

At the same time, competitive selection did not fully account for the stage-related findings. When analyses were restricted to the 35 finalists assessed at all three stages, significant within-athlete variation remained evident for mood disturbance, anxiety, stress, and recovery. Therefore, the overall pattern appears to reflect the coexistence of pre-existing between-athlete differences associated with subsequent qualification and within-athlete variation across successive pre-competition assessments. Importantly, the latter should not be interpreted as evidence that qualification itself caused psychological improvement, because time-varying influences related to training, recovery, competition preparation, and other contextual factors were not experimentally controlled.

Competitive progression in CrossFit® is inherently multifactorial. Qualification requires athletes to respond successfully to highly variable task demands and may be influenced by physiological and neuromuscular capacity, strength and power, work capacity, technical proficiency, movement efficiency, pacing strategies, competitive experience, previous performance, and familiarity with high-pressure competitive environments (11, 35–37). Psychobehavioral and recovery-related characteristics should therefore be interpreted as one component within this broader performance system. This interpretation is consistent with the ecological framework proposed by Luzes et al. (5), in which CrossFit® performance emerges from the interaction between performer characteristics and continuously changing task and environmental constraints. Accordingly, no single psychological, physiological, technical, or contextual characteristic should be expected to fully explain competitive progression.

The additional analyses of training background provide further context for these findings. Self-reported training experience, competitive experience, weekly training frequency, session duration, and number of daily training sessions did not consistently differ according to competitive progression and showed only weak associations with the principal psychobehavioral outcomes. Thus, the observed differences in mood, anxiety, stress, and perceived recovery do not appear to be readily explained by these broad indicators of training history or habitual training frequency.

However, this finding should not be interpreted as evidence that training demands were unrelated to psychobehavioral status. The variables available in the present study characterize training background rather than actual training load. Athletes reporting similar weekly training frequencies may differ substantially in session intensity, volume, density, modality distribution, periodization, and accumulated internal and external load (38–41). Consequently, unmeasured differences in competition preparation and training stress remain plausible contributors to the observed stress–recovery profiles.

Within this broader framework, lower mood disturbance, fatigue, anxiety, and perceived stress, together with higher perceived recovery, may characterize athletes presenting a more favorable pre-competition psychobehavioral profile. The stress–recovery relationship is particularly relevant to the present findings. Athletes represented at later competitive stages showed lower pre-competition stress-related scores and higher recovery-related scores. Moreover, within the Super Selective stage, recovery was the most consistent psychobehavioral dimension associated with subsequent qualification for the Finals. Rather than establishing recovery as an independent determinant of competitive success, these findings suggest that perceived recovery may provide complementary information regarding how athletes are coping with the combined demands of training and competition preparation. Previous evidence indicates that excessive stress and insufficient recovery may contribute to maladaptive responses, impaired performance, and increased injury risk, reinforcing the relevance of monitoring these variables during competitive periods (42).

State anxiety also showed an important relationship with competitive progression, although its association differed according to the analytical context. During the initial 4ALL assessment, athletes who subsequently qualified presented lower anxiety than those who were eliminated, and the multinomial regression model indicated that higher baseline anxiety was associated with lower odds of progressing to later competitive stages. However, anxiety did not significantly distinguish eventual finalists from eliminated athletes within the Super Selective stage after correction for multiple comparisons. Thus, anxiety may be particularly relevant for differentiating athletes within the broader initial competitive field, whereas recovery-related characteristics appeared more consistently associated with subsequent progression once the athlete pool became increasingly selected. This interpretation is consistent with evidence suggesting that elevated competitive anxiety may affect attentional control, decision-making, motor coordination, and technical execution under pressure (43).

Taken together, these findings support a multidimensional interpretation of psychobehavioral monitoring in competitive CrossFit®. Mood, anxiety, stress, and perceived recovery should not be interpreted as stand-alone indicators of competitive success but as complementary information that may be considered alongside training load, sleep, nutritional status, injury history, physiological capacity, technical performance, and competition-specific demands.

### 4.2 Interaction effects according to sex and competitive category

The interaction analyses suggested that biological sex had limited influence on psychobehavioral differences across competitive progression phases, whereas competitive category showed significant interactions for several mood, stress, and recovery variables. Only anger showed a significant sex-related interaction, suggesting that this specific emotional response may differ between men and women across competitive stages (44, 45). However, no significant sex-related interactions were observed for most psychobehavioral outcomes, including TMD, stress, recovery, and anxiety.

In contrast, competitive category showed significant interactions across multiple variables, including tension, anger, vigor, confusion, TMD, stress, and recovery. This suggests that athletes from different competitive categories may experience the season differently, possibly due to differences in competitive demands, training background, experience, psychological resources, and coping strategies. Previous evidence indicates that higher-level athletes often present more stable psychological profiles and greater emotional regulation capacity during decisive competitive situations (46, 47). Therefore, anger and TMD may be particularly sensitive indicators for identifying category-related differences in competitive stress and emotional regulation.

Overall, these findings suggest that competitive category may be more relevant than biological sex for explaining psychobehavioral differences across competitive stages. These findings indicate that the pattern of psychobehavioral responses across the competitive season was not completely homogeneous across official competition divisions. However, competitive category should not be interpreted as a direct measure of athletic expertise. Elite, Master, Teen, and Adapted divisions represent distinct competition classifications and may differ simultaneously in age, training history, physical capacity, competitive demands, previous experience, and other athlete-specific characteristics. The decision to retain these divisions within the primary analytical framework reflected the ecological objective of the study, which sought to characterize the athlete population participating in the actual national championship. Nevertheless, the significant phase × category interactions indicate that pooled stage effects should be interpreted as overall population-level patterns rather than as identical responses across all competitive groups.

Importantly, sensitivity analyses restricted to the Elite and Master divisions demonstrated that the main stage-related patterns for TMD, anxiety, global stress, and global recovery remained evident after Teen and Adapted athletes were excluded. Comparable directions of association were also observed when Elite and Master athletes were analyzed separately. These findings support the robustness of the principal results among the two largest competitive groups while also reinforcing the need for caution when generalizing these patterns to Teen and Adapted athletes. The small number of Teen and Adapted athletes who progressed to the final stage precluded reliable category-specific inferential modeling. Therefore, conclusions regarding these populations should be considered preliminary, and future studies should specifically recruit larger samples within these competitive divisions.

### 4.3 Baseline psychobehavioral characteristics associated with subsequent competitive progression

The results of the present study indicate that baseline psychobehavioral status was associated with competitive progression throughout the season. At baseline, athletes who subsequently progressed to later competitive stages exhibited lower levels of tension, depression, fatigue, anxiety, and total mood disturbance (TMD), alongside higher vigor and recovery-related scores. However, baseline general stress, specific stress, and global stress did not differ significantly between progression groups in the Kruskal–Wallis analyses. This distinction suggests that perceived recovery and mood-related variables showed stronger baseline differences according to subsequent competitive progression than stress-related scores when examined independently. Previous evidence supports the relevance of mood-state monitoring in athletes, showing that negative mood states and TMD are related to sport performance and may reflect changes in training load, fatigue, and competitive readiness (44, 48).

In particular, the largest ES was observed for global recovery, followed by TMD, general recovery, fatigue, specific recovery, and anxiety. This pattern suggests that competitive progression was not only associated with lower negative affective states, but also with a more favorable perceived recovery profile at baseline. Therefore, recovery-related variables may provide relevant complementary information regarding athletes’ pre-competition psychobehavioral and perceived-recovery status. However, they should not be interpreted in isolation as indicators of competitive readiness or subsequent performance. This interpretation is consistent with studies showing that perceived stress and recovery are closely related to mood states and may contribute to athlete monitoring during training and competition periods (49, 50).

The importance of baseline emotional state has been widely discussed in sport psychology. The “iceberg profile”, characterized by low negative mood states and high vigour, has traditionally been associated with superior sport performance. The present findings partially support this model, as athletes who reached the final stages presented a more favourable mood profile before the progression of the competition. Furthermore, fatigue and anxiety, which showed significant baseline differences between groups, may compromise attentional control, perceived effort, decision-making, and emotional regulation during high-pressure competitive situations (21, 51).

The multinomial regression analyses complemented these findings by showing that lower anxiety, lower TMD, lower global stress, and higher global recovery were associated with greater odds of competitive progression in the adjusted model. Although global stress did not differ significantly between groups in the univariate Kruskal–Wallis analysis, its association in the adjusted regression suggests that stress-related variables may still contribute to progression when considered together with anxiety, mood disturbance, recovery, and demographic covariates. Nevertheless, because this was an observational study and the regression model was exploratory, these variables should be interpreted as factors associated with competitive progression rather than definitive predictors of competitive success.

Although these associations remained evident in the adjusted multinomial model, they should not be interpreted as evidence of independent psychobehavioral determinants of qualification. The model adjusted for the variables available within the present dataset but could not comprehensively account for physiological performance, neuromuscular capacity, technical proficiency, movement efficiency, previous competitive success, or other sport-specific characteristics. Residual confounding is therefore plausible and should be considered when interpreting the magnitude and practical meaning of the associations.

Overall, these findings indicate that baseline psychobehavioral and recovery-related characteristics were associated with subsequent competitive progression. However, these measures should be interpreted within the broader multidimensional context of competitive performance and not as stand-alone indicators of qualification.

### 4.4 Exploratory nutritional and anthropometric findings

The nutritional analysis should be interpreted with caution. The regression model demonstrated low explanatory capacity and was not statistically significant, and none of the individual macronutrients showed a significant association with BMI. Therefore, these findings should be considered exploratory and hypothesis-generating rather than evidence of a specific relationship between recent macronutrient intake and BMI. In athletes, BMI is an imperfect proxy for adiposity because higher values may reflect greater fat-free mass rather than excess body fat, particularly in strength- and power-oriented populations such as CrossFit® athletes. Previous studies evaluating CrossFit® athletes have emphasized the relevance of body composition assessment beyond BMI, given the specific morphological and performance characteristics of this population (52).

Moreover, the use of a single self-reported 24-h dietary recall limits the ability to estimate habitual energy and macronutrient intake. The relatively low reported energy intake observed in the present sample may indicate dietary misreporting, day-to-day variability, or intentional short-term dietary strategies adopted during the competitive period. This issue is particularly relevant in athletic populations, in which energy expenditure and dietary practices may vary substantially according to training phase, competition schedule, recovery needs, supplement use, and performance-oriented nutritional behaviors. Recent evidence in CrossFit® nutrition highlights substantial heterogeneity in dietary practices, macronutrient distribution, and supplement use, reinforcing the need for more robust dietary assessment methods in future studies, such as repeated 24-h recalls, weighed food records, dietitian-assisted dietary analysis, or objective body-composition assessment (12, 24).

The absence of significant associations between carbohydrate, protein, and fat intake and BMI in the model may reflect the multifactorial nature of body mass regulation in trained athletes. Body mass and BMI in this context are influenced not only by dietary intake but also by training history, fat-free mass, hydration status, competitive category, energy expenditure, and periodized nutritional strategies. Importantly, total energy intake was retained as a descriptive characteristic of the sample but was not included simultaneously with the macronutrients in the revised regression model. In the initial analytical approach, its simultaneous inclusion produced substantial multicollinearity because total caloric intake is mathematically derived from carbohydrate, protein, and fat intake. Removing total energy from the multivariable model substantially reduced multicollinearity, with VIF values ranging from 1.38 to 1.68, while preserving the exploratory objective of examining macronutrient–BMI associations. Thus, future analyses should consider alternative nutritional approaches, including relative macronutrient intake normalized by body mass, energy availability, dietary patterns, and repeated dietary assessments across different phases of the competitive season.

### 4.5 Limitations

Several limitations should be considered when interpreting the present findings. First, psychobehavioral, training-related, anthropometric, and nutritional information was predominantly obtained through self-report and may therefore be affected by recall or response bias. Psychobehavioral assessments were standardized to a 7-day period preceding each competitive stage, although the exact interval between questionnaire completion and competition onset varied within this window. Nevertheless, questionnaires were completed before competition and not during, between, or immediately after workouts, reducing the influence of acute competition-related responses. In addition, the progressive elimination structure resulted in an increasingly selected sample across stages. To address this issue, complementary within-stage comparisons according to subsequent qualification status and a complete-case analysis restricted to finalists assessed across all three stages were performed, allowing selection effects to be distinguished from within-athlete variation.

Second, although several indicators of training background were available, including training and competitive experience, weekly training frequency, session duration, and daily training frequency, objective measures of internal and external training load were not collected. Therefore, differences in actual training volume, intensity, periodization, and accumulated physiological stress could not be fully accounted for when interpreting perceived stress and recovery. Similarly, objective measures of sleep, physiological strain, neuromuscular performance, and other markers of competition preparation were unavailable. The nutritional component was intentionally exploratory and relied on a single 24-h dietary recall, which represents recent rather than habitual intake. BMI was also derived from self-reported body mass and height and should not be interpreted as a direct measure of body composition.

Finally, the multinomial regression model was exploratory and was not externally validated. Although the temporal sequence of the pre-competition assessments and subsequent qualification strengthens the interpretation of the observed associations, causal or predictive conclusions cannot be established. Residual confounding also remains possible because relevant determinants of CrossFit® performance, including physiological capacity, strength and power, technical proficiency, pacing, movement efficiency, previous competitive performance, and familiarity with high-pressure environments, were not comprehensively quantified. Accordingly, the psychobehavioral and recovery-related variables identified in the present study should be interpreted as components associated with competitive progression within a multidimensional performance context rather than as independent determinants of qualification.

### 4.6 Practical applications

From an applied perspective, the present findings suggest that instruments assessing mood, state anxiety, perceived stress, and recovery may provide complementary information about athletes’ pre-competition psychobehavioral status during a competitive CrossFit® season. Such information may contribute to longitudinal athlete monitoring when interpreted together with objective indicators of training load, sleep, performance, injury status, and other competition-specific demands. However, these questionnaires should not be used in isolation to predict qualification or guide specific interventions. The present study did not evaluate predictive accuracy, clinical or performance-related decision thresholds, or whether questionnaire-guided interventions improve competitive outcomes. Their practical value should therefore be considered within a broader multidimensional monitoring framework. Nutritional monitoring should likewise be individualized and preferably supported by repeated dietary assessments conducted by qualified professionals.

### 4.7 Strengths and future recommendations

The present study has several strengths. First, it was conducted in a real-world competitive CrossFit® context, following athletes across an official national season composed of the online 4ALL qualifier, in-person Super Selective events, and national Finals. Second, the study integrated psychological, recovery-related, competitive, training, and nutritional variables, providing a multidimensional perspective on competitive progression. Third, the relatively large initial sample and the inclusion of different competitive categories increase the ecological relevance of the findings. Finally, the use of mixed models allowed the analysis of repeated assessments across competitive stages while accounting for within-participant dependency.

Future studies should expand this approach by including objective measures of training load, sleep, physiological strain, neuromuscular performance, hormonal and inflammatory markers, and body composition. Nutritional assessment should also be strengthened through repeated 24-h recalls, weighed food records, dietitian-assisted dietary analysis, and relative macronutrient intake normalized by body mass. In addition, future investigations should consider external validation of predictive models and the inclusion of larger samples within specific competitive categories, particularly teen and adapted athletes. These approaches may provide a more robust understanding of the interaction between psychobehavioral status, recovery, nutrition, and competitive performance in CrossFit® athletes.

## 5 Conclusions

In conclusion, CrossFit® athletes who progressed to later stages of a national competitive season presented more favorable psychobehavioral and recovery-related profiles, characterized by lower anxiety, lower mood disturbance, lower stress-related scores across competitive phases, and higher perceived recovery. Baseline anxiety, TMD, and global recovery were associated with competitive progression, while global stress was associated with progression in the adjusted regression model. These associations should not be interpreted as independent determinants of qualification, as competitive progression reflects the interaction of psychobehavioral, physiological, technical, training-related, and contextual characteristics. Nutritional variables showed limited explanatory capacity for BMI, and dietary findings should be interpreted cautiously due to the use of a single self-reported 24-h recall. Overall, these findings reinforce the importance of multidimensional athlete monitoring in CrossFit®, while highlighting the need for future studies integrating objective physiological, training-load, body-composition, and nutritional assessments.

## Data Availability

The data supporting the findings of this study are available from the corresponding author upon reasonable request, subject to ethical, confidentiality, and data-protection considerations, including compliance with the Brazilian General Data Protection Law (Lei Geral de Proteção de Dados Pessoais—LGPD; Law No. 13,709/2018).

## References

[1] Claudino JG Gabbett TJ Bourgeois F Souza HS Miranda RC Mezêncio B. Crossfit overview: systematic review and meta-analysis. Sports Med Open. (2018) 4:11. 10.1186/s40798-018-0124-5 29484512 PMC5826907

[2] Rios M Pyne DB Fernandes RJ. The effects of CrossFit® practice on physical fitness and overall quality of life. Int J Environ Res Public Health. (2025) 22:19. 10.3390/ijerph22010019 PMC11764515 39857472

[3] Prochnow T Amo C Patterson MS Heinrich KM. I CrossFit; do you? Cross-sectional peer similarity of physical activity behavior in a group high intensity functional training setting. Int J Environ Res Public Health. (2022) 19:4932. 10.3390/ijerph19094932 35564327 PMC9102180

[4] Martinho DV Rebelo A Gouveia ÉR Field A Costa R Ribeiro AS. The physical demands and physiological responses to CrossFit®: a scoping review with evidence gap map and meta-correlation. BMC Sports Sci Med Rehabil. (2024) 16:196. 10.1186/s13102-024-00986-3 39300545 PMC11414238

[5] De Oliveira Luzes TP De Almeida Garcia Moreira MA De Jesus Carrilho DF De Carvalho AQ. Exploring competition constraints at the CrossFit® games: a systematic characterisation through an ecological dynamics lens. J Sports Sci. (2026) 44:1554–64. 10.1080/02640414.2026.2653304 41923356

[6] D'alpino IA Moterosso JPC Botaro WR da Silva AOC Sant'anna PG Testa Junior A. Comparison between mood states, stress and recovery in CrossFit® competitors and non-competitors. J Phys Educ Sport. (2022) 22:2611–7. 10.7752/jpes.2022.11331

[7] Meier N Schlie J Schmidt A. Crossfit®: ‘unknowable’ or predictable?—a systematic review on predictors of CrossFit® performance. Sports. (2023) 11:112. 10.3390/sports11060112 37368562 PMC10304543

[8] Rios M Becker KM Monteiro AS Fonseca P Pyne DB Reis VM. Effect of the fran CrossFit workout on oxygen uptake kinetics, energetics, and postexercise muscle function in trained CrossFitters. Int J Sports Physiol Perform. (2024) 19:299–306. 10.1123/ijspp.2023-0201 38194958

[9] Butcher S Neyedly T Horvey K Benko C. Do physiological measures predict selected CrossFit® benchmark performance? Open Access J Sports Med. (2015) 241:241. 10.2147/oajsm.s88265 PMC4527742 26261428

[10] Dexheimer JD Schroeder ET Sawyer BJ Pettitt RW Aguinaldo AL Torrence WA. Physiological performance measures as indicators of crossfit® performance. Sports. (2019) 7:93. 10.3390/sports7040093 31013585 PMC6524377

[11] Leitão L Dias M Campos Y Vieira JG Sant'Ana L Telles LG. Physical and physiological predictors of FRAN CrossFit® WOD athlete's Performance. Int J Environ Res Public Health. (2021) 18:4070. 10.3390/ijerph18084070 PMC8069540 33921538

[12] Brisebois M Kramer S Lindsay KG Wu CT Kamla J. Dietary practices and supplement use among CrossFit® participants. J Int Soc Sports Nutr. (2022) 19:316–35. 10.1080/15502783.2022.2086016 35813850 PMC9261745

[13] Forte LDM Freire YGC Júnior JSDS Melo DA Meireles CLS. Physiological responses after two different CrossFit workouts. Biol Sport. (2022) 39:231–6. 10.5114/biolsport.2021.102928 35309530 PMC8919890

[14] Timón R Olcina G Camacho-Cardeñosa M Camacho-Cardenosa A Martinez-Guardado I Marcos-Serrano M. 48-hour recovery of biochemical parameters and physical performance after two modalities of CrossFit workouts. Biol Sport. (2019) 36:283–9. 10.5114/biolsport.2019.85458 PMC6786329 31624423

[15] Zanini GDS Pessôa Filho DM Neiva CM Da Silva DP Ciolac EG Verardi CEL. Stress and mood states monitoring in a swimming team during a competitive period. J Phys Educ Sport. (2018) 18:2466–71. 10.7752/jpes.2018.0436

[16] De Wet JS Recovery-stress states and training load of professional ballet dancers during a rehearsal and performance phase of a ballet year (2020)

[17] Maruschi MC Zanini GS Santorsula de Paula Oliveira PL Telles de Lima D Correa EA Verardi CEL. Psychosocial and body image variations in professional dancers: a prospective longitudinal observational study. Sports. (2026) 14:99. 10.3390/sports14030099 41893590 PMC13030549

[18] Brandt R Bevilacqua GG Crocetta TB D. M Monteiro CB Guarnieri R Hobold E. Comparisons of mood states associated with outcomes achieved by female and male athletes in high-level judo and Brazilian jiu-jitsu championships: psychological factors associated with the probability of success. J Strength Cond Res. (2021) 35:2518–24. 10.1519/JSC.0000000000003218 31453945

[19] Herring SA Ben Kibler W Putukian M Berkoff DJ Bytomski J Carson E. Load, overload, and recovery in the athlete: select issues for the team physician-A consensus statement. Curr Sports Med Rep. (2019) 18:141–8. 10.1249/JSR.0000000000000589 30969240

[20] Chang CJ Putukian M Aerni G Diamond AB Hong ES Ingram YM. Mental health issues and psychological factors in athletes: detection, management, effect on performance, and prevention: American Medical Society for sports medicine position statement. Clin J Sport Med. (2020) 30:E61–87. 10.1097/JSM.0000000000000817 32000169

[21] Bestwick-Stevenson T Toone R Neupert E Edwards K Kluzek S. Assessment of fatigue and recovery in sport: narrative review. Int J Sports Med. (2022) 43:1151–62. 10.1055/a-1834-7177 35468639

[22] Brenner JS Watson A Brooks MA Carl RL Briskin SM Canty G. Overuse injuries, overtraining, and burnout in young athletes. Pediatrics. (2024) 153:e2023065129. 10.1542/peds.2023-065129 38247370

[23] Bell L Ruddock A Maden-Wilkinson T Rogerson D. Overreaching and overtraining in strength sports and resistance training: a scoping review. J Sports Sci. (2020) 38:1897–912. 10.1080/02640414.2020.1763077 32602418

[24] Martinho DV Rebelo A Clemente FM Costa R Gouveia ÉR Field A. Nutrition in CrossFit®–scientific evidence and practical perspectives: a systematic scoping review. J Int Soc Sports Nutr. (2025) 22:2509674. 10.1080/15502783.2025.2509674 40471841 PMC12143013

[25] Margolis L Allen J Hatch-McChesney A Pasiakos S. Coingestion of carbohydrate and protein on muscle glycogen synthesis after exercise: a meta-analysis. Med Sci Sports Exerc. (2021) 53:384–93. 10.1249/mss.0000000000002476 32826640 PMC7803445

[26] Nielsen LLK Lambert MNT Jeppesen P. The effect of ingesting carbohydrate and proteins on athletic performance: a systematic review and meta-analysis of randomized controlled trials. Nutrients. (2020) 12:1483. 10.3390/nu12051483 32443678 PMC7284704

[27] Churchward-Venne T Pinckaers P Smeets J Betz M Senden J Goessens J. Dose-response effects of dietary protein on muscle protein synthesis during recovery from endurance exercise in young men: a double-blind randomized trial. Am J Clin Nutr. (2020) 112:303–17. 10.1093/ajcn/nqaa073 32359142 PMC7398777

[28] De S Zanini G De Oliveira DM De P Oliveira PLS Santos EC Da SA Bolzam R Hernández-Beltrán V. From performance to health: a global scientometric analysis of the evolution of CrossFit research. Sports. (2026) 14:213. 10.3390/sports14050213 42188589 PMC13210993

[29] Rohlfs ICPDM Rotta TM Luft CDB Andrade A Krebs RJ De Carvalho T. Brunel mood scale (BRUMS): an instrument for early detection of overtraining syndrome. Rev Bras Med Esporte. (2008) 14:176–81. 10.1590/S1517-86922008000300003

[30] McNair DM Lorr M Droppleman LF. Manual Profile of Mood States. San Diego, CA: Educational and Industrial Testing Service (1971).

[31] Kellmann M Kallus KW. Recovery-stress questionnaire for athletes. In: Kellmann M Kallus KW, editors. The Recovery-Stress Questionnaires. Abingdon: Routledge (2024). p. 76–136.

[32] Kellmann M Kallus KW Samulski D Costa L Simola R. Questionario de Estress e Recuperacao Para Atletas [The Recovery-Stress Questionnaire for Athletes]: Manual do Usuário. Belo Horizonte: Escola de Educação Física, Fisioterapia e Terapia Ocupacional, Universidade Federal de Minas Gerais (2009).

[33] Biaggio AMB Natalício L. Manual Para o Inventário de Ansiedade Traço-Estado (IDATE). Rio de Janeiro: CEPA (1979). p. 15.

[34] Amawi A AlKasasbeh W Jaradat M Almasri A Alobaidi S Hammad AA. Athletes’ nutritional demands: a narrative review of nutritional requirements. Front Nutr. (2024) 10:1331854. 10.3389/fnut.2023.1331854 38328685 PMC10848936

[35] Martínez-Gómez R Valenzuela PL Alejo LB Gil-Cabrera J Montalvo-Pérez A Talavera E. Physiological predictors of competition performance in CrossFit athletes. Int J Environ Res Public Health. (2020) 17:3699. 10.3390/ijerph17103699 PMC7277742 32456306

[36] Mangine GT Stratton MT Almeda CG Roberts MD Esmat TA VanDusseldorp TA. Physiological differences between advanced CrossFit athletes, recreational CrossFit participants, and physically-active adults. PLoS One. (2020) 15:e0223548. 10.1371/journal.pone.0223548 32255792 PMC7138313

[37] Ribeiro G De Aguiar RA Tramontin AF Martins EC Caputo F. Fatigue and performance rates as decision-making criteria in pacing control during CrossFit®. Percept Mot Skills. (2024) 131:1274–90. 10.1177/00315125241247858 38635574

[38] Maté-Muñoz JL Lougedo JH Barba M García-Fernández P Garnacho-Castaño MV Domínguez R. Muscular fatigue in response to different modalities of CrossFit sessions. PLoS One. (2017) 12:e0181855. 10.1371/journal.pone.0181855 PMC5533437 28753624

[39] Cavedon V Milanese C Marchi A Zancanaro C. Different amount of training affects body composition and performance in high-intensity functional training participants. PLoS One. (2020) 15:e0237887. 10.1371/journal.pone.0237887 32817652 PMC7440620

[40] Maté-Muñoz JL Lougedo JH Barba M Cañuelo-Márquez AM Guode-mar-Pérez J García-Fernández P. Cardiometabolic and muscular fatigue responses to different CrossFit® workouts. J Sports Sci Med. (2018) 17:668–79. Available online at: https://www.jssm.org/abstresearchajssm-17-668.xml.xml PMID: 30479537. PMC6243628 30479537

[41] Impellizzeri FM Marcora SM Coutts AJ. Internal and external training load: 15 years on. Int J Sports Physiol Perform. (2019) 14:270–3. 10.1123/ijspp.2018-0935 30614348

[42] Naderi A Shokri M Mokaberian M Tranaeus U. Understanding sports injury risks in CrossFit: a prospective cohort study on athletic demographics, training profiles, injury history, and psychological factors. Scand J Med Sci Sports. (2025) 35:e70100. 10.1111/sms.70100 40698870

[43] Tamminen KA Bonk D Milne M-J Watson JC. Emotion dysregulation, performance concerns, and mental health among Canadian athletes. Sci Rep. (2025) 15:2962. 10.1038/s41598-025-86195-5 39848997 PMC11758006

[44] Selmi O Ouergui I Muscella A Levitt DE Suzuki K Bouassida A. Monitoring mood state to improve performance in soccer players: a brief review. Front Psychol. (2023) 14:1095238. 10.3389/fpsyg.2023.1095238 36910823 PMC9992435

[45] Frank R Nixdorf I Beckmann J. Analyzing the relationship between burnout and depression in junior elite athletes. J Clin Sport Psychol. (2017) 11:287–303. 10.1123/JCSP.2017-0008

[46] Volgemute K Ulme G Abele A Vazne Z Ansons K Licis R. Multi-factorial profiling of athletes integrating personality, psychological skills, and psychophysiological performance indicators. Sci Rep. (2026) 16:4949. 10.1038/s41598-026-35809-7 41513758 PMC12877015

[47] Mitić P Nedeljković J Bojanić Ž Franceško M Milovanović I Bianco A. Differences in the psychological profiles of elite and non-elite athletes. Front Psychol. (2021) 12:635651. 10.3389/fpsyg.2021.635651 PMC8012685 33815222

[48] Lochbaum M Zanatta T Kirschling D May E. The profile of moods states and athletic performance: a meta-analysis of published studies. Eur J Investig Health Psychol Educ. (2021) 11:50–70. 10.3390/ejihpe11010005 34542449 PMC8314345

[49] Reynoso-Sánchez LF Pérez-Verduzco G Celestino-Sánchez MÁ López-Walle JM Zamarripa J Rangel-Colmenero BR. Competitive recovery–stress and mood states in Mexican youth athletes. Front Psychol. (2021) 11:627828. 10.3389/fpsyg.2020.627828 PMC7835411 33510698

[50] Gnacinski SL Meyer BB Wahl CA. Psychometric properties of the RESTQ-sport-36 in a collegiate student-athlete population. Front Psychol. (2021) 12:671919. 10.3389/fpsyg.2021.671919 34122264 PMC8187868

[51] De Miranda Rohlfs ICP Noce F Wilke C Terry VR Parsons-Smith RL Terry PC. Prevalence of specific mood profile clusters among elite and youth athletes at a Brazilian sports club. Sports. (2024) 12:195. 10.3390/sports12070195 39058086 PMC11280759

[52] Menargues-Ramírez R Sospedra I Holway F Hurtado-Sánchez JA Martínez-Sanz JM. Evaluation of body composition in CrossFit® athletes and the relation with their results in official training. Int J Environ Res Public Health. (2022) 19:11003. 10.3390/ijerph191711003 PMC9518485 36078716

