# Supplementary material for: Psychobehavioral profiles, competitive progression, and nutritional characteristics in CrossFit® athletes across a national competitive season
Source: Front Sports Act Living. 2026 Oct 6;8:1902121. doi: 10.3389/fspor.2026.1902121 (PMC13682298; doi:10.3389/fspor.2026.1902121)
Supplement: Supplementary file 1 [file Datasheet1.pdf]

Supplementary Table S1. Sensitivity analyses of the main psychobehavioral outcomes across competitive stages after restriction to Elite and Master athletes.

Competitive subset	Outcome	Super Selective vs 4ALL β (95% CI)	p	Finals vs 4ALL β (95% CI)	p	Overall phase p
**Elite + Master**	**TMD**	**−3.950 (−4.570 to −3.330)**	**<0.001**	**−6.452 (−7.452 to −5.452)**	**<0.001**	**<0.001**
	**State Anxiety**	**−5.943 (−7.627 to −4.259)**	**<0.001**	**−8.984 (−11.692 to −6.276)**	**<0.001**	**<0.001**
	**Global Stress**	**−0.106 (−0.126 to −0.086)**	**<0.001**	**−0.094 (−0.126 to −0.063)**	**<0.001**	**<0.001**
	**Global Recovery**	**0.092 (0.069 to 0.114)**	**<0.001**	**0.160 (0.124 to 0.196)**	**<0.001**	**<0.001**
**Elite**	**TMD**	**−3.978 (−4.715 to −3.241)**	**<0.001**	**−5.558 (−6.735 to −4.382)**	**<0.001**	**<0.001**
	**State Anxiety**	**−5.864 (−7.590 to −4.139)**	**<0.001**	**−9.314 (−12.061 to −6.567)**	**<0.001**	**<0.001**
	**Global Stress**	**−0.107 (−0.130 to −0.083)**	**<0.001**	**−0.122 (−0.159 to −0.084)**	**<0.001**	**<0.001**
	**Global Recovery**	**0.089 (0.057 to 0.122)**	**<0.001**	**0.144 (0.092 to 0.195)**	**<0.001**	**<0.001**
**Master**	**TMD**	**−3.916 (−4.913 to −2.919)**	**<0.001**	**−7.462 (−9.091 to −5.834)**	**<0.001**	**<0.001**
	**State Anxiety**	**−5.962 (−8.954 to −2.970)**	**<0.001**	**−8.395 (−13.266 to −3.525)**	**<0.001**	**<0.001**
	**Global Stress**	**−0.106 (−0.137 to −0.074)**	**<0.001**	**−0.063 (−0.114 to −0.012)**	**0.016**	**<0.001**
	**Global Recovery**	**0.094 (0.064 to 0.125)**	**<0.001**	**0.178 (0.128 to 0.227)**	**<0.001**	**<0.001**

Note. Sensitivity analyses were conducted using linear mixed models restricted to athletes competing in the Elite and Master divisions. Competitive stage was included as a fixed effect and participant identification as a random intercept. The 4ALL assessment was used as the reference stage. β values represent unstandardized fixed-effect coefficients, with negative values indicating lower scores and positive values indicating higher scores relative to 4ALL. Overall phase effects were evaluated using likelihood-ratio χ^2 tests.

TMD = Total Mood Disturbance; CI = confidence interval. Assessments were conducted within the seven days preceding each competitive stage. Statistical significance was set at $p < 0.05$

Supplementary Table S2. Within-stage psychobehavioral comparisons according to subsequent qualification status

Panel A. 4ALL: eliminated athletes vs athletes who subsequently qualified for Super Selective

Outcome	Eliminated after 4ALL (n=108)	Qualified for Super Selective (n=131)	U	Raw p	FDR-adjusted p	Cliff's δ
**Tension**	9 [7–10]	7 [5–9]	4923.0	<0.001	**<0.001**	−0.304
**Depression**	9 [7–11]	7 [5–9]	4927.5	<0.001	**<0.001**	−0.303
**Anger**	8 [6–10]	8 [6–10]	6664.5	0.440	0.456	0.058
**Vigor**	7 [5–9]	8 [6–11]	5301.0	<0.001	**0.001**	0.251
**Fatigue**	9 [7–11]	7 [5–9]	3783.0	<0.001	**<0.001**	−0.465
**Confusion**	8 [6–10]	8 [6–10]	6679.5	0.456	0.456	0.056
**TMD**	136 [131–139.25]	129 [124–135]	3953.5	<0.001	**<0.001**	−0.441
**General Stress**	2.04 [1.86–2.18]	1.96 [1.82–2.21]	6542.0	0.317	0.370	−0.075
**Specific Stress**	2.08 [1.83–2.33]	2.00 [1.75–2.33]	6110.5	0.070	0.089	−0.136
**Global Stress**	2.07 [1.93–2.23]	2.02 [1.87–2.19]	5973.0	0.039	0.054	−0.156
**General Recovery**	1.90 [1.70–2.10]	2.10 [1.95–2.30]	3927.5	<0.001	**<0.001**	0.445
**Specific Recovery**	1.81 [1.50–2.12]	2.12 [1.91–2.31]	4105.0	<0.001	**<0.001**	0.420
**Global Recovery**	1.86 [1.74–1.99]	2.11 [1.98–2.31]	2575.0	<0.001	**<0.001**	**0.636**

Outcome	Eliminated after 4ALL (n=108)	Qualified for Super Selective (n=131)	U	Raw p	FDR-adjusted p	Cliff's δ
**State Anxiety**	62.50 [42.75–70]	44 [32–56]	4477.5	<0.001	**<0.001**	−0.367

Panel B. Super Selective: eliminated athletes vs athletes who subsequently reached the Finals

Outcome	Eliminated after Super Selective (n=92)	Subsequent Finalists (n=35)	U	Raw p	FDR-adjusted p	Cliff's δ
Tension	7 [4–8]	7 [4.50–8]	1592.0	0.924	0.933	0.011
Depression	6 [5–9]	6 [4–8]	1498.0	0.545	0.763	−0.070
Anger	7 [5–10]	7 [5.50–9]	1587.5	0.905	0.933	0.014
Vigor	8.50 [6–11]	10 [7.50–12]	1206.0	0.029	0.087	0.251
Fatigue	6 [5–9]	5 [4.50–8]	1366.0	0.186	0.372	−0.152
Confusion	8 [6–10]	7 [5–9.50]	1329.0	0.128	0.299	−0.175
TMD	127.50 [121–132]	123 [119–129]	1210.0	0.031	0.087	−0.248
General Stress	1.92 [1.71–2.11]	1.86 [1.71–1.97]	1471.5	0.456	0.710	−0.086
Specific Stress	1.90 [1.63–2.19]	1.99 [1.65–2.22]	1594.0	0.933	0.933	−0.010
Global Stress	1.93 [1.74–2.06]	1.89 [1.72–2.10]	1559.5	0.787	0.933	−0.031
General Recovery	2.16 [2.00–2.33]	2.37 [2.15–2.59]	1131.5	0.010	**0.046**	0.297
Specific Recovery	2.15 [1.84–2.41]	2.36 [2.14–2.53]	1048.5	0.002	**0.017**	0.349

Outcome	Eliminated after Super Selective (n=92)	Subsequent Finalists (n=35)	U	Raw p	FDR-adjusted p	Cliff's δ
Global Recovery	2.14 [2.01–2.32]	2.33 [2.22–2.51]	798.5	<0.001	**<0.001**	**0.504**
State Anxiety	38.50 [28–53]	34 [23–52.50]	1435.5	0.346	0.606	−0.108

Note: Values are presented as median [interquartile range]. Between-group comparisons were performed using the Mann–Whitney U test. Reported U values correspond to the smaller of the two Mann–Whitney U statistics. P-values were adjusted across the 14 psychobehavioral outcomes within each competitive stage using the Benjamini–Hochberg false-discovery-rate (FDR) procedure. Cliff's δ was calculated with the subsequently qualified group as the reference direction; therefore, positive values indicate higher scores among athletes who subsequently qualified, whereas negative values indicate lower scores among subsequently qualified athletes. TMD = Total Mood Disturbance. Bold values indicate FDR-adjusted $p < 0.05$.

Supplementary Table S3. Complete-case within-athlete analysis among finalists assessed across all three competitive stages (n=35)

Outcome	4ALL	Super Selective	Finals	Friedman χ^2	p	FDR p: 4ALL vs SS	FDR p: 4ALL vs Finals	FDR p: SS vs Finals
Tension	7 [5–8.50]	7 [4.50–8]	6 [4–7]	21.220	<0.001	0.026	**<0.001**	**0.007**
Depression	7 [5–8.50]	6 [4–8]	6 [4–8.50]	14.233	<0.001	**0.002**	0.069	0.561
Anger	8 [6–10]	7 [5.50–9]	7 [5–9]	13.607	0.001	0.051	**0.011**	0.079
Vigor	10 [7–12]	10 [7.50–12]	10 [6.50–12]	8.990	0.011	**0.033**	0.104	0.818
Fatigue	6 [5–8]	5 [4.50–8]	5 [4–7]	23.170	<0.001	**0.041**	**0.002**	**0.035**
Confusion	7 [5–10]	7 [5–9.50]	7 [4–9]	21.216	<0.001	**0.008**	**<0.001**	0.182
TMD	126 [119.50–132]	123 [119–129]	123 [115.50–126]	**37.119**	<0.001	**<0.001**	**<0.001**	**0.005**
General Stress	1.93 [1.82–2.12]	1.86 [1.71–1.97]	1.81 [1.69–2.00]	17.371	<0.001	**<0.001**	**0.003**	0.777
Specific Stress	2.00 [1.75–2.17]	1.99 [1.65–2.22]	1.87 [1.67–2.17]	13.943	<0.001	**0.006**	**0.006**	0.955
Global Stress	1.99 [1.80–2.22]	1.89 [1.72–2.10]	1.88 [1.77–2.06]	**31.257**	<0.001	**<0.001**	**<0.001**	0.790
General Recovery	2.25 [2.10–2.45]	2.37 [2.15–2.59]	2.44 [2.20–2.59]	14.400	<0.001	**0.005**	**<0.001**	0.533

**Outcome**	**4ALL**	**Super Selective**	**Finals**	**Friedman χ²**	**p**	**FDR p: 4ALL vs SS**	**FDR p: 4ALL vs Finals**	**FDR p: SS vs Finals**
Specific Recovery	2.31 [2.12–2.47]	2.36 [2.14–2.53]	2.44 [2.30–2.65]	18.686	<0.001	0.062	**<0.001**	**0.013**
Global Recovery	2.31 [2.20–2.40]	2.33 [2.22–2.51]	2.45 [2.33–2.54]	**28.343**	<0.001	**0.004**	**<0.001**	**0.006**
State Anxiety	36 [28–55.50]	34 [23–52.50]	26 [20.50–44]	**29.829**	<0.001	**0.004**	**<0.001**	**0.019**

Note. Values are presented as median [interquartile range]. Analyses include only finalists who provided complete psychobehavioral assessments at all three competitive stages (n=35), thereby holding sample composition constant across time. Overall stage effects were tested using the Friedman test. When a significant overall effect was observed, pairwise comparisons were performed using Wilcoxon signed-rank tests, with Benjamini–Hochberg FDR correction applied across the three pairwise comparisons within each outcome. SS = Super Selective; TMD = Total Mood Disturbance. Bold values indicate FDR-adjusted $p < 0.05$.

Supplementary Table S4. Training-background characteristics according to competitive progression and their associations with principal psychobehavioral outcomes

Panel A. Distribution of training-background characteristics according to competitive progression

Training-background variable	Category	Eliminated after 4ALL (n=108)	Eliminated after Super Selective (n=92)	Finalists (n=39)	χ^2 (df)	p	Cramer's V
**CrossFit® training experience**	0–1 year	14 (13.0%)	14 (15.2%)	4 (10.3%)	**7.182 (10)**	**0.708**	**0.123**
	2–3 years	22 (20.4%)	17 (18.5%)	6 (15.4%)			
	4–5 years	16 (14.8%)	18 (19.6%)	9 (23.1%)			
	6–7 years	16 (14.8%)	13 (14.1%)	9 (23.1%)			
	8–9 years	22 (20.4%)	13 (14.1%)	3 (7.7%)			
	>10 years	18 (16.7%)	17 (18.5%)	8 (20.5%)			
**Competitive experience**	0–1 year	17 (15.7%)	21 (22.8%)	4 (10.3%)	**13.680 (10)**	**0.188**	**0.169**
	2–3 years	19 (17.6%)	20 (21.7%)	5 (12.8%)			
	4–5 years	16 (14.8%)	15 (16.3%)	9 (23.1%)			
	6–7 years	23 (21.3%)	15 (16.3%)	5 (12.8%)			
	8–9 years	13 (12.0%)	13 (14.1%)	10 (25.6%)			
	>10 years	20 (18.5%)	8 (8.7%)	6 (15.4%)			

Training-background variable	Category	Eliminated after 4ALL (n=108)	Eliminated after Super Selective (n=92)	Finalists (n=39)	χ² (df)	p	Cramer's V
**Weekly training frequency**	3–4 days/week	12 (11.1%)	18 (19.6%)	8 (20.5%)	**7.119 (4)**	**0.130**	**0.122**
	5–6 days/week	82 (75.9%)	70 (76.1%)	27 (69.2%)			
	7 days/week	14 (13.0%)	4 (4.3%)	4 (10.3%)			
**Typical session duration**	<60 min	10 (9.3%)	7 (7.6%)	1 (2.6%)	**8.145 (6)**	**0.228**	**0.131**
	61–90 min	27 (25.0%)	31 (33.7%)	17 (43.6%)			
	91–120 min	51 (47.2%)	44 (47.8%)	14 (35.9%)			
	>121 min	20 (18.5%)	10 (10.9%)	7 (17.9%)			
**Daily training sessions**	1 session/day	44 (40.7%)	51 (55.4%)	19 (48.7%)	**4.319 (2)**	**0.115**	**0.134**
	2 sessions/day	64 (59.3%)	41 (44.6%)	20 (51.3%)			

Panel B. Complementary ordinal comparisons across-progressions groups

Training-background variable	Kruskal–Wallis H	p	FDR-adjusted p
CrossFit® training experience	0.303	0.860	0.860
Competitive experience	6.760	**0.034**	0.124
Weekly training frequency	6.010	**0.050**	0.124
Typical session duration	1.598	0.450	0.562
Daily training sessions	4.301	0.116	0.194

Panel C. Associations between training-background characteristics and baseline psychobehavioral outcomes

Training-background variable	Psychobehavioral outcome	Spearman ρ	p	FDR-adjusted p
**CrossFit® training experience**	TMD	0.008	0.904	0.998
	State anxiety	−0.000	0.998	0.998
	Global Stress	0.021	0.744	0.930
	Global Recovery	0.034	0.603	0.927
**Competitive experience**	TMD	0.112	0.083	0.830
	State anxiety	0.002	0.977	0.998
	Global Stress	−0.046	0.483	0.927
	Global Recovery	0.030	0.641	0.927
**Weekly training frequency**	TMD	−0.027	0.678	0.927
	State anxiety	0.029	0.658	0.927
	Global Stress	**0.145**	**0.025**	**0.508**
	Global Recovery	−0.067	0.305	0.927
**Typical session duration**	TMD	0.072	0.267	0.927
	State anxiety	0.007	0.912	0.998
	Global Stress	−0.059	0.363	0.927
	Global Recovery	−0.069	0.287	0.927

Training-background variable	Psychobehavioral outcome	Spearman ρ	p	FDR-adjusted p
**Daily training sessions**	TMD	0.051	0.436	0.927
	State anxiety	−0.025	0.696	0.927
	Global Stress	0.046	0.478	0.927
	Global Recovery	−0.031	0.633	0.927

Note. Values in Panel A are presented as n (%) within each competitive-progression group. Competitive progression groups were defined according to the furthest stage reached during the 2025 TCB season: eliminated after 4ALL (n = 108), eliminated after Super Selective (n = 92), and finalists (n = 39). Cramer's V was used as the effect-size measure for categorical comparisons. Panel B presents complementary analyses in which ordered training-background categories were analyzed using Kruskal–Wallis tests; p-values were adjusted across the five comparisons using the Benjamini–Hochberg false-discovery-rate (FDR) procedure. Panel C presents Spearman rank-order correlations between training-background variables and psychobehavioral outcomes assessed at the initial 4ALL pre-competition assessment (n = 239); FDR adjustment was applied across the 20 correlations. TMD = Total Mood Disturbance; FDR = false discovery rate.
